# Subcompartmentalization by cross-membranes during early growth of *Streptomyces* hyphae

**DOI:** 10.1038/ncomms12467

**Published:** 2016-08-12

**Authors:** Paula Yagüe, Joost Willemse, Roman I. Koning, Beatriz Rioseras, María T. López-García, Nathaly Gonzalez-Quiñonez, Carmen Lopez-Iglesias, Pavel V. Shliaha, Adelina Rogowska-Wrzesinska, Abraham J. Koster, Ole N. Jensen, Gilles P. van Wezel, Ángel Manteca

**Affiliations:** 1 Área de Microbiología, Departamento de Biología Funcional e IUOPA, Facultad de Medicina, Universidad de Oviedo, 33006 Oviedo, Spain; 2 Molecular Biotechnology, Institute of Biology, Leiden University, Sylviusweg 72, P.O. Box 9502, 2300RA Leiden, The Netherlands; 3 Department of Molecular Cell Biology, Leiden University Medical Centre, PO Box 9600, 2300RC Leiden, The Netherlands; 4 Crio-Microscòpia Electrònica. Centres Científics i Tecnològics, Universitat de Barcelona, 08028 Barcelona, Spain; 5 Department of Biochemistry and Molecular Biology and VILLUM Center for Bioanalytical Sciences, University of Southern Denmark, Campusvej 55, DK-5230, Odense M, Denmark; 6 Microbial Ecology, Netherlands Institute for Ecology (NIOO-KNAW), PO Box 50, 6700AB Wageningen, The Netherlands

## Abstract

Bacteria of the genus *Streptomyces* are a model system for bacterial multicellularity. Their mycelial life style involves the formation of long multinucleated hyphae during vegetative growth, with occasional cross-walls separating long compartments. Reproduction occurs by specialized aerial hyphae, which differentiate into chains of uninucleoid spores. While the tubulin-like FtsZ protein is required for the formation of all peptidoglycan-based septa in *Streptomyces*, canonical divisome-dependent cell division only occurs during sporulation. Here we report extensive subcompartmentalization in young vegetative hyphae of *Streptomyces coelicolor*, whereby 1 μm compartments are formed by nucleic acid stain-impermeable barriers. These barriers possess the permeability properties of membranes and at least some of them are cross-membranes without detectable peptidoglycan. Z-ladders form during the early growth, but cross-membrane formation does not depend on FtsZ. Thus, a new level of hyphal organization is presented involving unprecedented high-frequency compartmentalization, which changes the old dogma that *Streptomyces* vegetative hyphae have scarce compartmentalization.

## Abstract (web summary)

Bacteria of the genus *Streptomyces* form cellular filaments (hyphae) in which sporadic peptidoglycan cell walls separate multinucleate compartments. Here, Yagüe *et al*. show that young hyphae are further compartmentalized by cross-membranes lacking detectable peptidoglycan.

## Graphical abstract

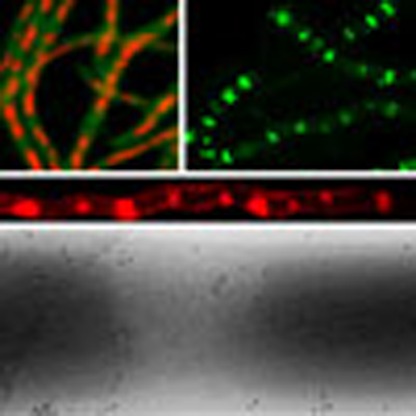

Streptomycetes are filamentous Gram-positive bacteria that are of great importance for biotechnology given their ability to produce a large array of natural products, including antibiotics, anticancer agents and immunosuppressants, as well as a plethora of industrial enzymes^1^,^2^.

The *Streptomyces* life cycle has largely been studied during the growth of surface-grown cultures^3^,^4^,^5^,^6^,^7^,^8^ (Fig. 1). The life cycle starts with the germination of a spore, which expands out via tip growth and hyphal branching to form a vegetative mycelium consisting of multinucleate compartments^3^. When dispersal is required, for example, after nutrient depletion, the vegetative mycelium eventually differentiates into a new so-called aerial mycelium, which grows into the air. The aerial hyphae are also initially multinucleated, but these eventually develop sporogenic structures that differentiate into chains of unigenomic spores^4^. The lysis of the substrate mycelium and, later, the early aerial mycelium, exhibits the hallmarks of programmed cell death (PCD), with the involvement of specific lytic enzymes (nucleases, proteases and muramidases)^5^,^6^. New features affecting early development have been described over the last decade^7^,^8^. An early compartmentalized mycelium (MI) undergoes an early PCD-like process affecting the substrate and aerial hyphae^6^. Remarkably, live and dying cells are alternately observed in the MI hyphae^6^. The lifespan of this young mycelium is very short under laboratory conditions, but it is likely the predominant mycelium in cultures grown under natural conditions, such as in non-amended soils^7^. The substrate and aerial hyphae in the MII phase^6^ are physiologically different from those in the MI phase. MI corresponds to the vegetative mycelium, whereas the substrate and aerial mycelia are the reproductive stages driving towards sporulation^8^. Secondary metabolism is typically restricted to the MII phase^8^.

The study of cell division in *Streptomyces* has primarily focused on sporulation-specific cell division, in which ladders of Z-rings are formed, resulting in chains of spores^4^. In contrast, during vegetative growth, the hyphae are compartmentalized by occasional cross-walls, which delimit adjacent elongated compartments containing multiple copies of the chromosome. Consequently, streptomycetes are rare examples of multicellular bacteria^9^,^10^. During both sporulation and vegetative growth, the GTPase FtsZ polymerizes to form a dynamic ring-like structure known as the Z-ring^11^,^12^. Surprisingly, in the model organism *Streptomyces coelicolor* A3(2), *ftsZ* is required for cell division (and thus for sporulation) but not for growth, and the deletion of *ftsZ* results in hyphae that are devoid of septa^13^. The process of vegetative cell division differs mechanistically from canonical division, as illustrated by the fact that many of the other canonical cell division genes (for example, *ftsI, ftsL* and *ftsW*) are required for sporulation but not for cross-wall formation^14^,^15^. Recently, a novel mechanism of cell division was established in vegetative hyphae of *Streptomyces*, based on membranous structures instead of peptidoglycan-based cross-walls^16^.

So far, the mechanisms of cell division and hyphal compartmentalization during early vegetative growth (MI stage) have been poorly characterized. Here we provide insights into the ultrastructure of MI hyphae, the regulation of MI compartmentalization, and the kinetics of membrane permeability alteration during PCD in *Streptomyces*. Our data also reveal a surprisingly high frequency of subcompartmentalization of the MI hyphae by cross-membranes.

## Results

### MI hyphae exhibit differential membrane permeability

**Figure 2:**
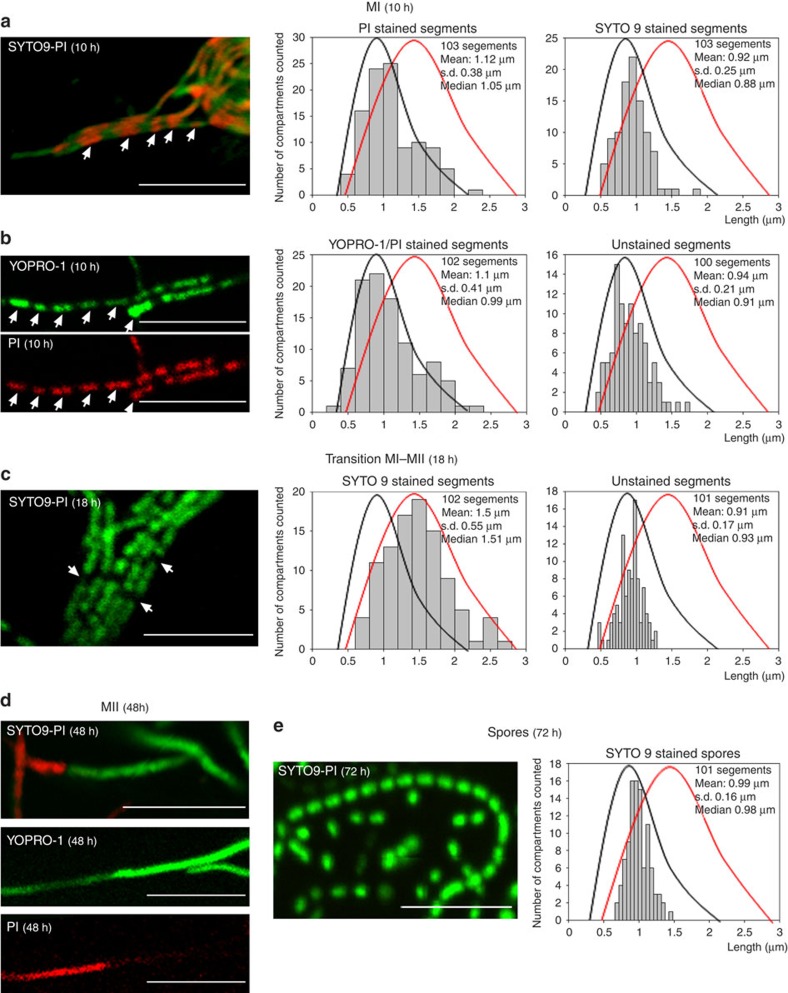
Confocal laser scanning fluorescence microscopy analysis of *S. coelicolor* growing on GYM agar. (**a**) SYTO9 (green) and PI (red) staining (MI, 10 h). (**b**) YOPRO-1 (green) and PI (red) staining (MI, 10 h). (**c**) SYTO9-PI staining (transition from MI to MII, 18 h). (**d**) SYTO9-PI staining and YOPRO-1-PI (MII, 48 h). YOPRO-1 and PI were usezd simultaneously; notice that not all of the hyphae stained with YOPRO-1 were stained with PI. (**e**) SYTO9-PI staining (spores, 72 h). The scale bars correspond to 8 μm. The arrows in **a**–**c** highlight dying cells in ‘MI' and ‘transition MI-MII' hyphae. Histograms of the stained and unstained segments are shown. Two distributions were observed: one from the MI stained and unstained segments in **a** and **b** and from the unstained segments in **c** (black lines), and the second from the living segments stained with SYTO9, which begin to enlarge as multinucleated hyphae in **c** (red lines).

PCD leads to changes in the membrane permeability of cells and to the alternation of propidium iodide (PI) membrane-permeable and non-permeable cellular segments (Fig. 2a) in the same continuous hyphae^6^ (compare fluorescence and phase-contrast images in Supplementary Fig. 1). The average size of MI segments demonstrating differences in permeability was 1.1±0.38 and 0.9±0.25 for PI- and SYTO9-stained segments, respectively, and compartmentalization affected 100% of the analysed hyphae (Fig. 2a; Supplementary Fig. 1). In addition to SYTO9 and PI, we used the non-permeable nucleic acid stain YOPRO-1, which selectively stains eukaryotic apoptotic cells^17^. YOPRO-1 permits the visualization of cells with altered selective permeability that have not yet undergone complete lysis and are therefore not stained by PI^18^. We applied this new technique here to determine whether partial lysis (as observed in eukaryotic apoptotic cells) also occurs in *Streptomyces*. Cells that were stained with PI were also stained with YOPRO-1 (Fig. 2b), whereas live cells were not stained (compare Fig. 2a with Fig. 2b). However, when the samples were processed for microscopy in a more rapid manner (within seconds instead of minutes), the dying cells in the MI hyphae were stained only with YOPRO-1 and not with PI (Supplementary Fig. 2), resembling eukaryotic apoptosis. The average size of the segments stained with SYTO9, PI, and YOPRO-1 at the MI stage was 1.1±0.41 μm (Fig. 2a,b), similar to the spacing observed during sporulation-specific compartmentalization (0.99±0.16 μm) (Fig. 2e). At later time points (transition MI-MII, 18 h), SYTO9-stained cells (live cells) grew as multinucleated compartments, and the average compartment size increased from 1.0 μm (Fig. 2a,b, curves in black) to 1.5±0.55 μm (Fig. 2c, curves in red). Dying cells were no longer stained with either PI or YOPRO-1, most likely due to the complete DNA degradation, but compartmentalization was still observed, and the average length of these unstained segments (18 h; average size of 0.91±0.17 μm) (Fig. 2c) was the same as that observed in early dying cells stained with PI (Fig. 2a,b). The regular pattern of cellular segments with differential, alternating permeabilities to PI and YOPRO-1 in the same hyphae indicates the existence of permeability barriers to these two vital stains separating cellular segments in the MI hyphae.

A regular pattern of permeable and non-permeable cells was not observed during the MII stage, once the dying MI cells disintegrated^6^, while live cells grew out to form non-septated multinucleated hyphae (Fig. 2d). This indicates that the regular pattern of YOPRO-1/PI staining observed in the MI hyphae is attributable to the nature of this mycelium, which differs from that of the MII hyphae.

During the last developmental stages, the hyphae compartmentalized into spores with an average diameter of 0.99±0.16 μm and a size distribution comparable to that of the MI segments (compare Fig. 2a–c with Fig. 2e). Sporulating septa consisted of very thick cell walls that were not stained by nucleic acid-binding stains and were observed as unstained regions (Fig. 2e; notice that the length of the unstained regions corresponding to thick cell walls is much shorter than that of the unstained regions corresponding to MI cellular segments).

### Visualization of compartmentalization by electron microscopy

**Figure 3:**
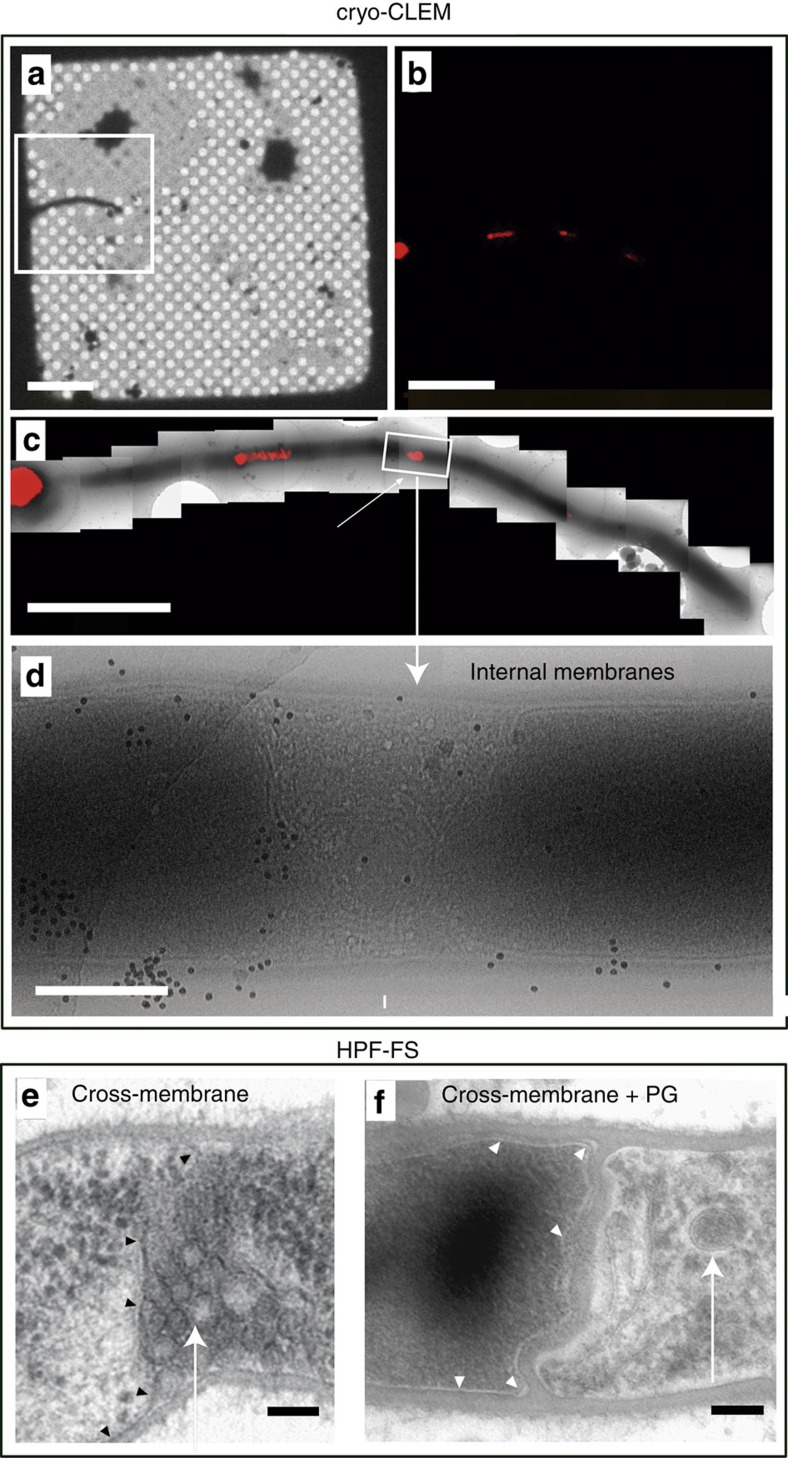
Cryo-correlative light and electron microscopy (cryo-CLEM) and high-pressure freezing and freeze substitution (HPF-FS) of 12-hour MI hyphae of *S. coelicolor* grown on GYM agar. (**a**–**d**) Cryo-CLEM. (**a**) Phase-contrast mode, (**b**,**c**) FM5-95 (red) staining, (**d**) electron microscopy. (**e**,**f**) HPF-FS electron microscopy, (**e**) cross-membrane without cell wall, (**f**) cross-membrane with a thick cell wall. Arrows indicate internal cross-membranes in the form of membrane vesicles and membrane arrays. Arrowheads indicate cross-membranes continuous with the extracellular membrane delimiting cellular segments. Scale bars: (**a**) 20 μm, (**b**) 5 μm, (**c**) 4 μm, (**d**) 500 nm, (**e**) and (**f**) 100 nm.

To obtain detailed insight into the discontinuities along the MI mycelium (12 h), the hyphae were analysed by performing cryo-correlative light/electron microscopy (cryo-CLEM; Fig. 3a–d) and high-pressure freezing and freeze substitution electron microscopy (Fig. 3e,f). Two types of cells/compartments were observed to alternate and contained either weakly FM5-95-stained and electron-dense cytoplasm, or strongly FM5-95-stained and electron-lucent cytoplasms (Fig. 3b–d). In the latter, vesicles and membrane invaginations were frequently observed (arrows in Fig. 3). Samples for cryo-CLEM were flash-frozen within milliseconds in liquid ethane, without chemical fixation, minimizing the possibility that the membranous structures observed may have been the result of chemical artifacts^18^,^19^.

Two types of barriers delimiting MI cellular segments were detected: cross-membranes without detectable peptidoglycan under the tested conditions (Fig. 3e); and peptidoglycan-based cross-walls (Fig. 3f).

### FtsZ expression and Z-ring formation in MI hyphae

**Figure 4:**
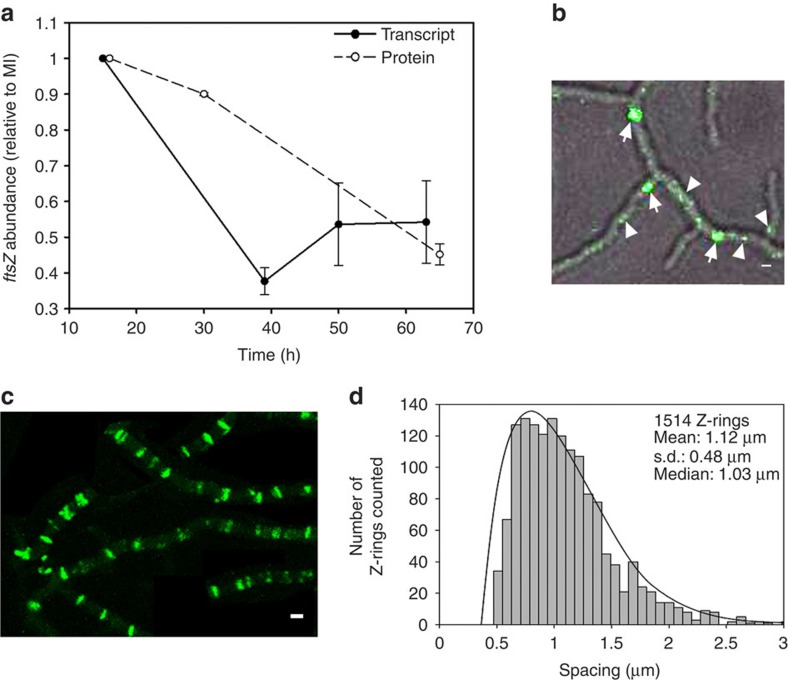
*ftsZ* gene expression, protein abundance and cellular localization. (**a**) qRT–PCR analysis of *FtsZ* mRNA (solid line) and FtsZ protein abundance (dashed line). The average values of three biological replicates are presented (with SD). The MI sample (15 h in transcriptomics, 16 h in proteomics) was used for normalization and consequently has a value of 1 and an s.d. of 0. All abundance values were significantly different with respect to the 15-h sample (*P* value<0.05; limma analysis^43^ for protein; analysis of variance with Turkey's HSD *post hoc* analysis for transcript). (**b**) Z-ring formation shown by eGFP-FtsZ at early developmental time points of *S. coelicolor* grown on GYM agar plates (12 h). Fluorescence and phase-contrast images are overlaid. Arrowheads label transitory dynamic spiral-like structures that do not cross the entire diameter of the hyphae. Arrows label Z-rings. (**c**) Maximum projection of the time-lapse experiments (15 h); 100% of the hyphae had a regular pattern of Z-rings as indicated by eGFP-FtsZ (see Supplementary Movie 1 and Supplementary Fig. 1). (**d**) Spacing of the Z-rings. The spacing of a total of 1514 Z-rings was analyzed. Scale bars in **b** and **c** correspond to 1 μm.

MI is a transitory stage in laboratory cultures, and has been ignored in most studies examining *Streptomyces* development. However, previous transcriptome analysis of RNA isolated from young hyphae of *S. coelicolor* suggested that *ftsZ* is overexpressed at this stage of the life cycle^8^. We performed quantitative reverse transcription–PCR (qRT–PCR) analysis of RNA isolated from solid-grown cultures on GYM agar plates to show that *ftsZ* transcript levels are higher after spore germination (MI, 15 h) and are even higher those observed during sporulation (that is, 63–70 h; solid line in Fig. 4a). The lowest *ftsZ* transcript levels were observed at ∼39 h, corresponding to the formation of multinucleated substrate/pre-sporulating aerial hyphae. *ftsZ* gene expression correlates well with FtsZ protein abundance (dashed line Fig. 4a), as quantified by tandem mass tag (TMT) protein labelling and LC-MS/MS. FtsZ was more abundant at the MI stage than during sporulation.

There is a strong correlation between the frequency of septation—and thus compartment sizes—and the expression level of FtsZ: high levels of FtsZ are required to support sporulation-specific cell division^20^,^21^. Therefore, we examined whether high *ftsZ* transcription and protein levels also correspond to the septation frequency during the earliest stages of growth after spore germination. The cellular localization of FtsZ-eGFP was analysed by performing confocal microscopy with *S. coelicolor* FM145, a derivative of the model strain M145 exhibiting low autoflurorescence^22^ (Fig. 4b,c; Supplementary Fig. 1 and Supplementary Movie 1). Z-ring formation begins with the development of dynamic spiral-like structures of FtsZ, which are visualized as transitory spots that move rapidly inside the mycelium, and do not cross the entire diameter of the hypha^11^,^12^ (arrowheads in Fig. 4b, Supplementary Movie 1). These spots ultimately form Z-rings, which are more stable and cross the entire diameter of the hypha (arrows in Fig. 4b, Supplementary Movie 1). During the early MI stage, Z-rings formed asynchronously during MI growth (Supplementary Movie 1). Z-rings disappear once the septa are complete^23^,^24^; consequently, they could not be observed at the same developmental time point in a single image (Supplementary Movie 1). However, the maximum projection of images acquired during an overnight time-lapse experiment (Fig. 4c and Supplementary Fig. 1) revealed that the Z-rings in the Z-ladders were spaced at an average of 1.1±0.48 μm in all of the MI hyphae (Fig. 4d and Supplementary Fig. 1). This spacing and regularity are highly similar to that observed during sporulation-specific cell division^24^.

To analyse the relationship between the Z-rings observed in *S. coelicolor* FM145 expressing FtsZ-eGFP as well as the differences in the PI permeability observed in the MI, both techniques were combined, staining the *S. coelicolor* FM145 strain expressing FtsZ-eGFP with PI. Z-rings are transitory, and PI permeability barriers can only be visualized when dying cells (stained with PI) alternate with living cells (not stained with PI). Consequently, it was difficult to detect Z-rings at a discrete time point coinciding with the borders between a dying and a living cell (Supplementary Fig. 3). The colocalization of Z-rings with PI permeability barriers suggests that at least some of the Z-rings observed at the MI stage may contribute to the formation of permeability barriers separating PI permeable/impermeable segments.

### Membrane permeability of the *ftsZ* mutant

**Figure 5:**
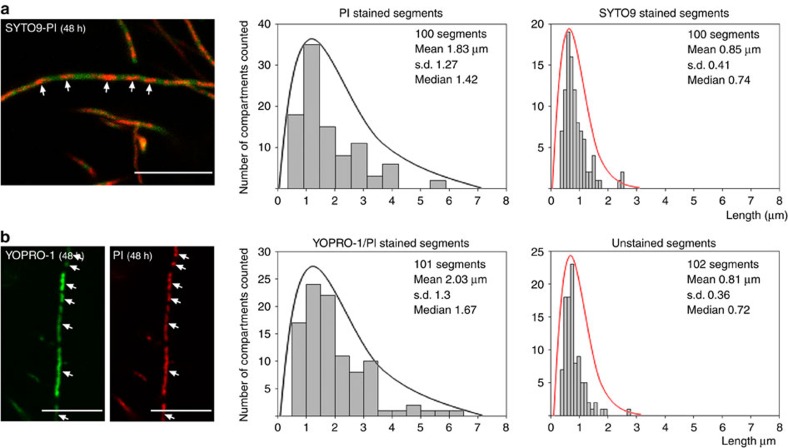
Confocal laser scanning fluorescence microscopy analysis of the *ftsZ* mutant HU133. (**a**) SYTO9 (green) and PI (red) staining (48 h). (**b**) YOPRO-1 (green) and PI (red) staining (48 h). Histograms of the stained and unstained segments are shown. Two distributions were observed: one from viable segments stained with SYTO9 and not stained with YOPRO-1 or PI (red lines); the second from dying cells stained with YOPRO-1 and/or PI (black lines). The scale bars correspond to 8 μm.

SYTO9/PI and YOPRO-1/PI staining were applied to the *ftsZ* null mutant HU133 (McCormick *et al*.^13^). Surprisingly, the *ftsZ* mutant exhibited an alternating pattern of PI/YOPRO-1 permeable and impermeable segments comparable to that of the parental strain (Fig. 5). This pattern was observed at all time points in the mutant (the images shown in Fig. 5 correspond to a 48-h culture). The average size of the live segments, that is, those stained with SYTO9 but not with PI or YOPRO-1, was 0.85 μm±0.41 and 0.81 μm±0.36, respectively (red curves in Fig. 5), comparable to the sizes observed in the parental strain (see above and Fig. 2). As discussed below, the average length of dying cells, thta is, those stained with PI and YOPRO-1, was 1.83 μm±1.27 and 2.03 μm±1.3, respectively, with a maximum length of 6.12 μm (curves in black in Fig. 5), which was double the length observed in the parental strain. This pattern of PI/YOPRO1-permeable and -impermeable segments alternating in the same hypha was present in 100% of the mycelium (Supplementary Fig. 4).

### Membrane and cell wall staining of *Streptomyces* hyphae

**Figure 6:**
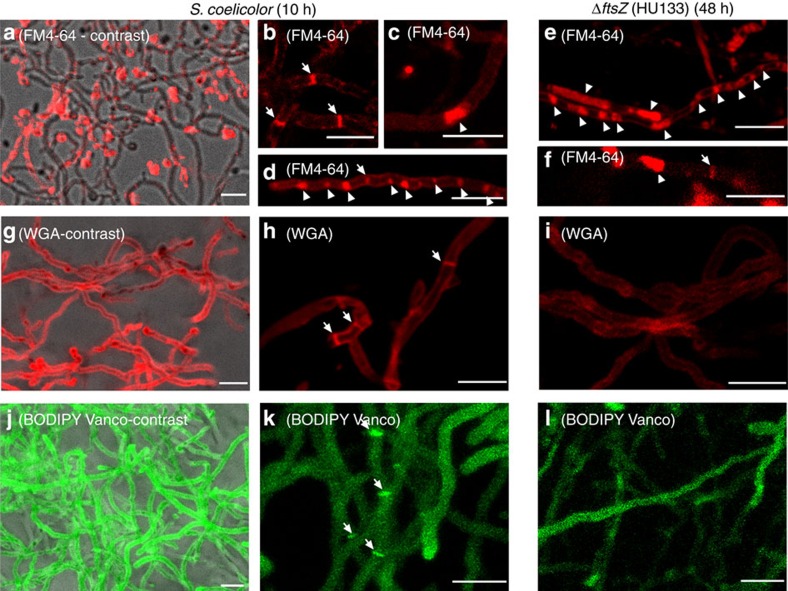
Membrane and cell wall staining of *S. coelicolor* and its *ftsZ* mutant HU133. (**a**–**f**) FM4-64 staining (membranes). (**g**–**i**) WGA staining (cell wall). (**j**–**l**) BODIPY-vancomycin staining (nascent peptidoglycan). Fluorescent images in **a**, **g** and **j** correspond to the maximum projection 10-μm series overlaid with their respective phase-contrast images, showing 100% of the stained hyphae. Arrows indicate cross-membranes and cross-cell walls. Arrowheads indicate membrane cellular segments filled with membrane vesicles. Scale bars, 4 μm.

The lipophilic membrane colourant FM4-64 was used to stain hyphae of *S. coelicolor* M145 at the MI stage (Fig. 6a–d), and this staining was compared with HU133 (*ftsZ* mutant; Fig. 6e,f). As previously reported^7^, FM4-64 stained the *S. coelicolor* hyphae heterogeneously, and only a fraction of the hyphae were stained (compare the hyphae observed by phase-contrast with those stained with FM4-64 in Fig. 6a). Two types of internal membranes were detected: sharp cross-membranes continuous with the extracellular membrane and delimiting cellular segments (arrows in Fig. 6b) and large spots stained with FM4-64 (arrowhead in Fig. 6c). At the MI stage, some hyphae exhibited a regular pattern of cross-membranes, and/or FM4-64 stained spots (Fig. 6d), but this pattern was not observed in all hyphae. As discussed below, FM4-64 could not be used to quantify the proportion of cross-membranes in the MI hyphae, because it does not stain all membranes under the conditions employed in this work. FM4-64 also stained internal membranes in the *ftsZ* null mutant (Fig. 6e,f). The two types of internal membranes described above were observed, but with an obvious difference: the large spots stained with FM4-64 were much larger in the *ftsZ* null mutant than in the parental strain (compare Fig. 6c with Fig. 6e).

Cell wall stains such as fluo-wheat germ agglutinin (fluo-WGA) and boron-dipyrromethene-vancomycin (BODIPY-vancomycin) stained 100% of the hyphae observed (Fig. 6g,j). Fluo-WGA stained the complete external hyphal walls and the cross-walls of the MI septa in the *S. coelicolor* parental strain (Fig. 6h). BODIPY-vancomycin stains nascent peptidoglycan^25^, and most of the cell walls were not visualized with this stain (Fig. 6k,l). D-amino acid pulse labelling of cell walls^26^ gave the same results as those observed for fluo-WGA staining (Supplementary Fig. 5). The frequency of cross-walls stained with all cell wall colourants was lower than the frequency of the PI and YOPRO-1 permeability barriers as previously described (Fig. 2). Specifically, most of the permeability barriers separating PI/YOPRO-1-permeable and -impermeable segments do not have sufficient cell wall to be observed by fluorescence microscopy. The use of fluorescent D-amino acids combined with PI *in vivo* showed that septa (membranes with thick cell walls^15^) colocalize only with some of the PI permeability barriers (Supplementary Fig. 3). This provides further evidence that, at minimum, the PI permeability barriers colocalizing with cross walls in the MI correspond to cross-membranes. Fluo-WGA, fluorescent D-amino acids or BODIPY-vancomycin did not stain cross walls in the *ftsZ* null mutant (Fig. 6i,l), as expected for a mutant without cross walls^13^.

*S. coelicolor* FM145 expressing FtsZ-eGFP was stained with FM4-64 (membrane stain) and HADA (cell wall stain), which indicated that at least a portion of the Z-rings colocalize with cross-walls and/or cross-membranes (Supplementary Fig. 3). As discussed below, Z-rings are transitory, and FM4-64 does not stain all membranes, but colocalization can be detected, providing further evidence that Z-rings may be involved in the formation of cross-membranes.

### Compartmentalization correlates with protoplast formation

**Figure 7:**
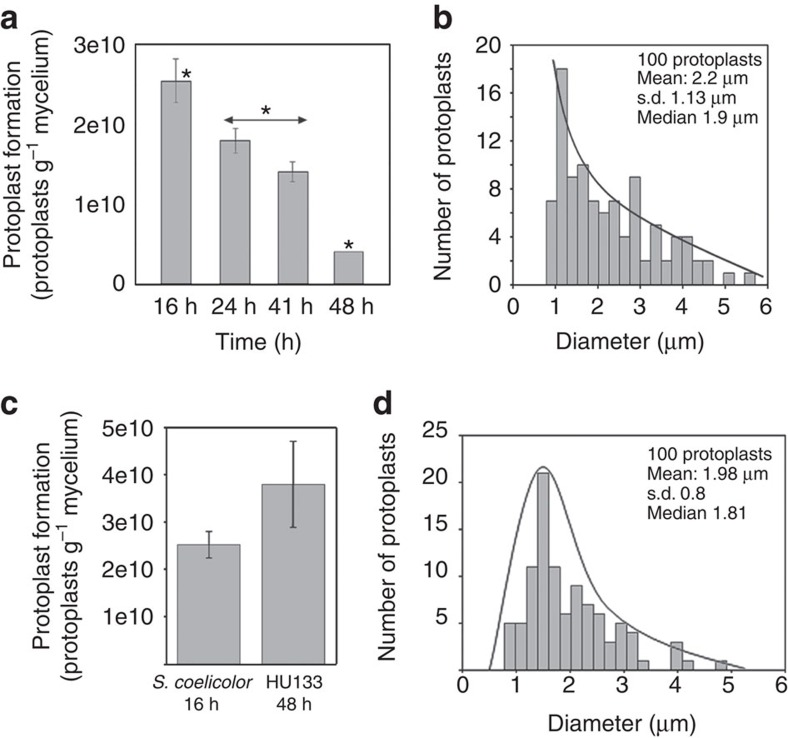
Protoplast formation correlates with MI and compartmentalization in the *ftsZ* mutant HU133. (**a**,**b**) Protoplast formation in the *S. coelicolor* parental strain (presented as the number of protoplasts per gram of fresh mycelium) grown in GYM and a histogram of protoplast diameter at 10 h. Significant differences in protoplast formation (*P* value<0.05; analysis of variance with Turkey's HSD *post hoc* analysis) with respect to the 16 h sample (MI) are labelled with an asterisk. (**c**,**d**) Protoplast formation in the Δ*FtsZ* HU133 mutant at 48 h of growth in GYM and histograms of protoplast diameter. The error bars indicate±s.d. of three biological replicates.

The ability to form protoplasts depends on the differentiation stage^27^, and this feature can be used to distinguish MI-compartmentalized hyphae from MII-multinucleated hyphae because MII hyphae do not form many protoplasts, likely due to the instability of the large protoplasts formed by multinucleated hyphae^6^. We devised a method based on protoplast formation and flow cytometry measurements to quantify the number of protoplasts formed per unit of biomass. Protoplasts formed in high amounts during the MI stage (16 h), whereas their numbers progressively decreased during the MI and MII transition phase, and very few protoplasts were produced at the late MII stage (48 h) (Fig. 7a). The average protoplast size was 2.2±1.13 μm, and there were no protoplasts with diameters larger than 5–6 μm (Fig. 7b; Supplementary Fig. 6). During the sporulation stages, unigenomic spores were readily obtained, but no protoplasts were observed (data not shown) because *Streptomyces* spores are resistant to lysozyme^28^. Protoplast formation correlated well with the compartmentalization observed in the MI but not MII hyphae (see above), thus providing a method to assess the degree of compartmentalization of the hyphae and to distinguish between the MI and MII phases.

The *ftsZ* null mutant (*S. coelicolor* HU133) formed protoplasts in numbers and with an average diameter (1.98±0.8; Fig. 7c,d; Supplementary Fig. 6) comparable to those of the *S. coelicolor* parental strain at the MI stage. The *S. coelicolor ftsZ* null mutant grows very slowly, and its growth was therefore not comparable to the parental strain. *S. coelicolor* HU133 formed protoplasts at all time points (the protoplasts quantified in Fig. 7c,d were obtained from a 48-h culture).

## Discussion

**Figure 8:**
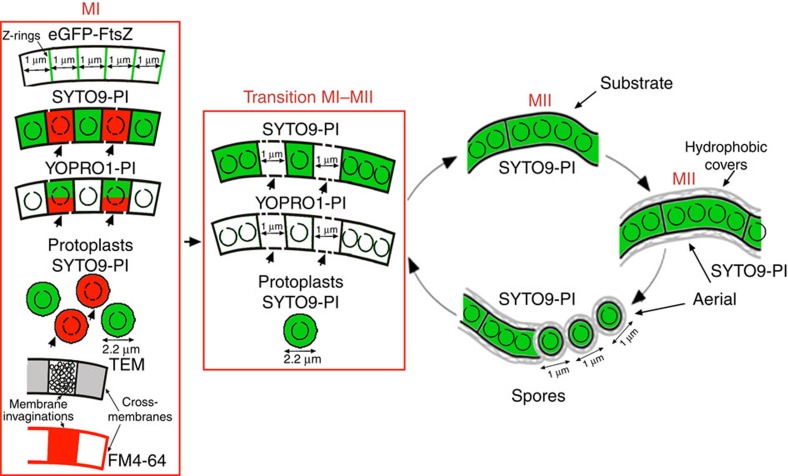
Model of compartment formation and PCD in vegetative hyphae of *Streptomyces coelicolor*. Z-rings, cross-membranes, membrane invaginations/vesicles, and protoplasts are illustrated. Peptidoglycan walls (not shown in the scheme) are associated with some of the cross-membranes forming classical septa. Open circles inside compartments represent intact chromosomal DNA, and fragmented circles indicate degraded chromosomal DNA. Membrane discontinuities represent the changes in membrane permeability in dying cells. Red corresponds to PI fluorescence, and green corresponds to SYTO9 or YOPRO1 fluorescence. Arrows indicate dying cells; DNA is fully degraded in these cells during the transition from MI to MII and thus is not stained. FM4-64 labelling is illustrated in red.

The alternation of PI/YOPRO1-permeable/impermeable segments in the MI hyphae demonstrates the existence of barriers impermeable to these viability stains, with the diffusion properties of membranes^6^ (outlined in Fig. 8). The frequency of these permeability barriers is much higher than the frequency of septa formed by cross walls^3^. Here we applied cryo-CLEM and FM lipophilic styryl dyes (FM4-64/FM5-95) to reveal the existence of two types of internal membranes, which were not associated with detectable peptidoglycan: cross-membranes continuous with the extracellular membrane that delimit cellular segments and are difficult to be observed (Fig. 3 and Fig. 6b); and vesicles/membrane arrays, that are non-continuous with the extracellular membrane, and are easily visualized by CLEM (Fig. 3 and Fig. 6c). As discussed below, cross-membranes are also found in the *ftsZ* null mutant, which is not able to produce peptidoglycan-based septa^13^. Importantly, recent FRAP and CLEM/cryo-electron tomography experiments performed on liquid-grown mycelia, revealed the existence of impermeable cross-membranes compartmentalizing vegetative hyphae of *Streptomyces albus*, and their existence was also corroborated in *S. coelicolor*^16^. In the current work, experimentation was performed at the earliest stages of growth (MI stage) in solid-grown cultures of *S. coelicolor*, with the experiments aimed at quantifying the nature and the extent of hyphal compartmentalization. Our fluorescence and electron microscopy experiments failed to detect 1-μm spacing cross-membranes correlating with the 1-μm spacing permeability barriers observed with PI/YOPRO-1. This is most likely explained by the fact that fluorescence and electron microscopy do not permit the visualization of all *Streptomyces* membranes, as it happens in other microorganisms. For example, FM4-64 only stains vacuolar membranes in yeast^29^, the inner membrane and membrane domains enriched in basic phospholipids in *E. coli*^30^, the outer membrane in *Agrobacterium*^31^ and under the conditions employed in this work, only a fraction of *S. coelicolor* hyphae (Fig. 6a). Next-generation electron microscopy methodologies, such as CLEM and cryo-electron tomography, are enabling the detection of novel internal structures in bacteria, including membranes, but the existence of further undetected structures cannot be dismissed (reviewed in Jensen *et al*.^32^). Interestingly, the use of fluorescent D-amino acids combined with PI *in vivo* showed that the membranes associated with cross walls (septa), can be detected as PI permeability barriers colocalizing with cross walls (Supplementary Fig. 3). PI permeability barriers that are not associated with cross walls most likely also represent cross-membranes. The compartmentalization of MI hyphae correlates with the ability to form stable protoplasts, which again supports the existence of cross-membranes surrounding the compartments that are able to produce protoplasts (Fig. 7a,b). Further work employing new and improved microscopy techniques will be necessary to test whether all permeability barriers to PI and YOPRO-1 correspond to membranes.

In contrast to the cross-membranes delimiting cellular segments, internal membranous structures are easier to contrast against the cytoplasm of the MI hyphae, and were described long ago in *S. coelicolor* by Glauert and Hopwood^33^ and in dying cells of *S. antibioticus* by Miguelez *et al*^5^. However, these authors applied chemical fixation, and thus they could not discard the possibility that these structures were chemical artifacts^5^; to this day, their discovery has remained unvalidated. Further work is necessary to characterize the biological function of vesicles/membrane invaginations in the MI hyphae, but their observation by cryo-electron microscopy supports the conclusion that they are not chemical artifacts. Celler *et al*.^16^ recently observed internal membranous structures in vegetative hyphae using cryo-electron tomography and CLEM. The authors showed that large membrane assemblies are formed creating DNA-free zones, often (but not always) associated with initiation of septum formation, suggesting a role of these structures in protecting the DNA during the onset of vegetative cell division.

Two different types of septa exist in *Streptomyces*, both of which consist of peptidoglycan and membranes: the cross walls in the substrate and early aerial mycelia and the sporulation septa in sporulating aerial hyphae. Although their formation depends on FtsZ, the localization of the Z-rings is regulated by entirely different mechanisms during these two growth phases^14^,^15^,^34^,^35^. The compartmentalization of MI hyphae is comparable to that of sporulation-specific cell division, with the formation of ladders of Z-rings, with an average spacing of 1 μm. A proportion of the Z-rings observed in the MI colocalize with cross-membranes, and some colocalize with PI permeability barriers (Supplementary Fig. 3). These results indicate that Z-rings might contribute to the formation of at least some of the permeability barriers separating the 1-μm cellular segments and again suggest that PI permeability barriers correspond to the cross-membranes observed by CLEM. One of the most intriguing peculiarities of *Streptomyces* cell division is that *ftsZ* null mutants are viable, resulting in long non-septated branched vegetative hyphae^13^. Counterintuitively, the hyphae of the *ftsZ*-null mutant can be fragmented without loss of viability, suggesting that the release of their contents is somehow prevented^13^. Here we have provided evidence for the existence of cross-membranes in the *ftsZ*-null mutant (Fig. 6e,f). The formation of membranous- rather than peptidoglycan-based ‘septa' that are not dependent on FtsZ is so far unique in bacteria. Interestingly, a somewhat analogous case is known in archaea: most Crenarchaea lack *ftsZ*, but some such as *Pyrobaculum islandicum*, produce cross-walls that consist of an S-layer rather than peptidoglycan^36^. Further work should characterize the role of FtsZ (if any) in the formation of cross-membranes and identify any possible differences between the cross-membranes formed in the presence or absence of *ftsZ*.

In summary, this work provides evidence for the existence of an unprecedented high-frequency compartmentalization in MI hyphae based on cross-membranes. Cross-membranes may have developed to support the multicellular life style of streptomycetes, enabling subcompartmentalization to provide an additional level of organization in the long hyphae. It will be very interesting to determine whether similar membrane-based compartmentalization also exists in other (multicellular) bacteria.

## Methods

### Strains and media

*Streptomyces coelicolor* M145 (ref. 37) was obtained from the John Innes Centre strain collection and its *ftsZ* null mutant HU133 (ref. 13) was obtained from the Harvard University strain collection. GYM (glucose, yeast and malt)^38^ was used as the growth medium in both liquid and solid media. Agar plates were used with and without cellophane disks and were inoculated with 100 μl of an inoculum suspension (1 × 10^7^ viable spores ml^−1^), followed by incubation at 30 °C. The reduced autofluorescence strain *S. coelicolor* FM145 harbouring a plasmid expressing FtsZ-eGFP was previously described^22^.

### Real-time qRT–PCR

Total RNA was obtained by phenol extraction and using the RNeasy Midi Kit (Qiagen). RNA integrity was verified using a 2100 BioAnalyzer (Agilent). The RNAs used in real-time RT–PCR analysis were digested with the TURBO DNA-free kit (Ambion) to remove possible DNA contamination according to the manufacturer's instructions. Briefly, 50 μl of 200 ng μl^−1^ RNA solution was treated with DNase I at 37 °C for 30 min. The samples were mixed with 0.2 volumes of inactivation reagent, incubated for 5 min at room temperature and recovered by centrifugation.

One microgram of RNA was used as the template for complementary DNA (cDNA) synthesis using the High-Capacity cDNA Reverse Transcription Kit (Applied Biosystems) according to the manufacturer's specifications. The primers used for real-time PCR of *ftsZ* were 5′- GCAGCACCGCAGAACTAC -3′ and 5′- AGACCGACCTCGATCATCC -3′. Real-time PCR was performed on an ABI PRISM 7900 HT thermocycler (Applied Biosystems). The reactions contained 2 μl of cDNA diluted twofold, 10 μl of SYBR Green PCR Master Mix (Applied Biosystems) and 300 nM primers in a final volume of 20 μl. Three biological samples were analysed, and control reactions with RNA and water as templates were performed to verify the absence of DNA contamination and primer–dimer formation. The thermal profile was as follows: an initial stage at 50 °C for 2 min, a second stage at 95 °C for 10 min, a third stage of 40 cycles at 95 °C for 15 s and 60 °C for 1 min, and a final dissociation profile of 95 °C for 15 s, 60 °C for 15 s and 95 °C for 15 s to confirm the absence of primer dimers. Relative quantification of gene expression was performed using the ΔΔCt method^39^. *SCO3878*, which encodes the β-chain of DNA polymerase III, was used as an internal control to quantify the relative expression of the target gene employing experimental conditions as previously reported^8^. *SCO3878* was expressed constitutively under the conditions used in this work^8^. The reliability of the differences in the *ftsZ* expression levels was analysed by analysis of variance with Turkey's honest significant difference (HSD) *post hoc* analysis. Differences were considered as significant if their *P* value was ≤0.05.

### FtsZ protein quantification

Protein was extracted from *Streptomyces* cultures at 16, 30 and 65 h as previously described^40^. The 30- and 65-h samples were analysed in biological triplicate, and the 16-h samples were analysed in quadruplicate. Protein pellets were resuspended in 8 M urea with 50 mM TEAB for bicinchoninic acid assay quantitation. A 300-μg quantity of protein was digested using a combined trypsin/LysC digestion protocol^41^. The samples were then desalted and subjected to amino acid analysis. For each condition, 60 μg of peptides was labelled with 0.5 mg of TMT-10-plex reagent (ThermoFisher) following the manufacturer's protocol. The samples were then combined, and 50 μg was fractionated by hydrophilic interaction liquid chromatography to generate 12 fractions, which were further fractionated on an EasyLC system (Thermo) with a 90-min gradient (0–35%). The LC aqueous mobile phase contained 0.1% (v/v) formic acid in water, and the organic mobile phase contained 0.1% (v/v) formic acid in 95% (v/v) acetonitrile. The samples were injected on a custom 3-cm trap column (100-μM internal diameter silica tubing packed with Reprosil 120 C18 5-μM particles) and desalted with 18 μl of buffer A. Separation was performed on a custom 20-cm column (75-μM internal diameter silica tubing packed with Reprosil 120 C18 3-μM particles) with a pulled emitter at 250 nl min^−1^. The eluted peptides were analysed on an Orbitrap Fusion mass spectrometer in data-dependent mode. The MS1 spectrum was acquired on an Orbitrap mass analyser at 120,000 resolution with an AGC target of 5e5. For MS2 scans, peptides were isolated with a quadrupole using a 1.2-Da isolation window and fragmented at 35 and 40% normalized collision energy. AGC was set at 5e4, the maximum injection time was 120 ms and dynamic exclusion was 20 s. Data were processed with Proteome Discoverer 2.1 with Mascot as the search engine using the UniProt *S. coelicolor* database (retrieved on 06.03.15). Peptides were validated by Mascot Percolator with a threshold of 0.01 PEP. Peptide spectrum matches with total summed reporter intensities of <4e5 were not considered for quantitation due to the high level of noise in the quantitation data. The quantification results of peptide spectrum matches were converted to peptide-level quantitation, which in turn was converted into protein quantitation using an R script^42^. Only proteins with two or more quantified peptides were considered. This resulted in quantitation of 3,575 proteins (manuscript in preparation). FtsZ was quantified by 13 peptides (Supplementary Table 1). Proteins were analysed for differential expression using the limma package in R^43^. *P* values were adjusted for multiple comparison using the R stats package. Thus the reported *Q* values for the change in FtsZ expression were adjusted for multiple comparisons within the data set. The relative abundance (normalized as the ratio against the 16 h sample) was estimated using the TMT abundances from three biological replicates (Supplementary Table 1).

### Viability staining

Culture samples were obtained and processed as previously reported^6^ for excised cellophane or agar pieces and were stained a few minutes later. The LIVE/DEAD BacLight Bacterial Viability Kit (Invitrogen, L-13152) was employed for staining. This kit uses SYTO9 and PI, two DNA-binding colourants. SYTO9 penetrates intact membranes and stains viable cells green, whereas PI only penetrates bacteria with damaged membranes. At the concentrations used in the kit, PI displaces SYTO9 from DNA when both colourants are present in dying cells, staining cells red^44^. Samples were observed under a Leica TCS-SP2-AOBS and/or Leica TCS SP8 laser scanning microscope at wavelengths of 488 and 568 nm for excitation and 530 (green) or 630 nm (red) for emission. More than 100 images were analysed in a minimum of three independent culture analyses for each developmental condition.

Both YOPRO-1 (Invitrogen Y3603) and PI were used at concentrations of 5 μM. YOPRO-1 was observed by confocal microscopy using the same parameters described above for SYTO9.

Images were processed using Image J software. The lengths of at least 100 stained and unstained segments were quantified, and histograms of length distributions and statistical analyses were constructed using SigmaPlot 12.0.

### Membrane staining

The lipophilic styryl dye, N-(3-triethylammoniumpropyl)-4-(p-diethylaminophenyl-hexatrienyl) pyridinium dibromide (FM4-64) (Molecular Probes, T-3166) was added directly to the culture medium at a final concentration of 1 mg ml^−1^ before the plates were poured. This concentration of FM4-64 does not affect growth. Samples were observed under a confocal laser scanning microscope at wavelengths of 550 nm for excitation and 700 nm for emission.

### Cell wall staining

Cells were fixed for 15 min at room temperature using PBS (0.14 M NaCl, 2.6 mM KCl, 1.8 mM KH_2_PO_4_ and 10 mM Na_2_HPO_4_) containing 2.8% paraformaldehyde and 0.0045% glutaraldehyde. Texas Red WGA (Invitrogen W21405) was added at a concentration of 100 mg ml^−1^ in 2% BSA in PBS and the cells were incubated at room temperature for 3 h. BODIPY-vancomycin (Invitrogen V34850) was used at a concentration of 0.5 μg ml^−1^ in PBS for 15 min. The samples were washed with PBS and observed under a Leica TCS-SP8 confocal laser scanning microscope at excitation wavelengths of 595/505 and emission wavelengths of 615/513 for WGA and BODIPY-vancomycin, respectively.

Fluorescent D-alanine (HADA) was used as recommended by Kuru *et al*.^26^. Briefly, the HADA stock solution was prepared in dimethylsulphoxide at a concentration of 100 mM. In the case of the *S. coelicolor* parental strain, liquid cultures (0.5 ml) containing 500 μM HADA were inoculated with fresh spores (10^7^ spores per ml) and incubated at 200 r.p.m.s. and 30 °C. The *ftsZ* null mutant did not grow in liquid cultures under our experimental conditions; instead it was grown in small (0.5 ml) solid GYM cultures with 500 μM HADA.

### Time-lapsed live imaging

The spores were initially incubated on GYM medium, for six hours. Samples were then excised out and inverted into uncoated m-dishes (Ibidi GmbH). The lids were turned so they were supported on the vents to allow gas exchange and were then sealed with two layers of parafilm to prevent drying of the medium. The samples were incubated at 30 °C and imaged with a Zeiss Observer confocal microscope. Images were acquired every 45 min for 15 h. Excitation was performed with a 488-nm laser, and detection was performed with a 505–530 nm bandpass filter. To minimize focal drift, the microscope stage and imaging chamber were allowed to equilibrate for 60 min before imaging.

Time-lapse images were processed with ImageJ. Z-rings were detected using a Gaussian filter with a Sigma value of 2 followed by an Unsharp Mask filter with a radius of 3 and a mask weight of 0.6. Then, a Find Maxima process with a noise tolerance of 50 was used to obtain binary images of the Z-ring local maxima. Finally, the nearest distance between Z-rings was calculated using the Nearest Neighbourhood Distance plugin (Nnd; https://icme.hpc.msstate.edu/mediawiki/index.php/Nearest_Neighbor_Distances_Calculation_with_ImageJ).

### Cryo-correlative light and electron microscopy

For cryo-CLEM, an EM grid was positioned on a *Streptomyces* culture during growth and was vitrified directly afterwards by plunging into liquid ethane using a Leica EM GP from RT at approximately 75% humidity with 1-second blotting. Plunge-frozen grids were used for correlative light and microscopy. Plunge-frozen EM grids containing *Streptomyces* were imaged using a fluorescence microscope equipped with CMS196 cryo light microscope stage (Linkam, Surrey, UK), in conjunction with a Zeiss Axio Imager M2. Cryo-EM was performed on a Tecnai 20 FEG operated at 200 kV (FEI Company). Images were recorded on a 2k × 2k camera mounted behind a GIF energy filter (Gatan) operated at a slit width of 20 eV.

### High-pressure freezing and freeze substitution

To observe *S. coelicolor* cells by transmission electron microscopy (TEM), Epon-embedded thin sections were obtained as described by Frias *et al*.^45^. Briefly, bacterial cells were cryo-immobilized as quickly as possible using a Leica EMPact high-pressure freezer (Leica, Vienna, Austria). Frozen samples were freeze-substituted in a Leica EM automatic freeze substitution system (Leica, Vienna, Austria). The substitution was performed in pure acetone containing 2% (wt/vol) osmium tetroxide and 0.1% (wt/vol) uranyl acetate at 90 °C for 72 h. The temperature was gradually decreased (5 °C h^−1^) to 4 °C, held constant for 2 h, and then finally increased to room temperature and maintained for 1 h. The samples were washed for 1 h in acetone at room temperature and infiltrated in a graded series of Epon-acetone mixtures: 1:3 for 2 h, 2:2 for 2 h, 3:1 for 16 h, and pure Epon 812 (Ted Pella, Inc.) for 30 h. The samples were embedded in fresh Epon and polymerized at 60 °C for 48 h. Ultrathin sections were cut with a Leica UCT ultramicrotome and mounted on Formvar carbon-coated copper grids. Sections were post-stained with 2% (wt/vol) aqueous uranyl acetate and lead citrate and examined with a Tecnai Spirit electron microscope (FEI Company, The Netherlands) at an acceleration voltage of 120 kV.

### Mycelium protoplasting

Protoplasts were obtained according to the method described by Okanishi *et al*.^27^, and Kieser *et al*.^37^ with some modifications to ensure that the efficiency of protoplasting was close to 100% (as analysed by observing the disintegration of the hyphae by phase-contrast and confocal microscopy, Supplementary Fig. 6) and that there was no significant loss of protoplasts during manipulations, both of which are critical requirements for reproducible and significant flow cytometry measurements (see below).

Mycelia grown on cellophane discs were scraped off, and 60 mg of mycelia (fresh weight) were resuspended in 1.12 ml of buffer P (0.6% TES buffer pH 7.2, 103% sucrose) in a 2-ml Eppendorf tube. Lysozyme was added from a freshly prepared stock at a final concentration of 2 mg ml^−1^ and incubated for 30 min at 600 r.p.m. and 37 °C in an Eppendorf ThermoMixer. Protoplasts were drawn in and out twice in a 1-ml pipette, incubated for an additional 30 min, washed two times by sedimentation (1,000*g*) and resuspended in buffer P. After the final wash, the protoplasts were resuspended in 500 μl of buffer P.

The original buffer P described by Kieser *et al*.^37^ included a trace element solution and other salts. These salts interfere with flow cytometry measurements, and consequently, it was necessary to use the modified buffer P described in this work, which only includes TES buffer and sucrose (K_2_SO_4_, MgCl_2_ and the trace element solution were not added). The buffer P used in these experiments was filtered through a 0.2-μm filter.

### Protoplast quantification

Protoplast samples were stained with SYTO9 and quantified directly by fluorescence microscopy using a Thoma chamber (depth: 0.02 mm) or a flow cytometer (Cytomics FC500, Beckman-Coulter, Inc., Miami, FL, USA). In all cases, protoplasts from two biological replicates were quantified. Both methodologies produced similar results, but only flow cytometry data were included in this work.

Flow cytometry measurements were performed using BD Trucount Tubes (reference 340334), containing 500 μl of protoplasts stained with SYTO9 (6 μM). The trigger signal was established with an FL1 detector (530/540 nm) with an adequate negative control (buffer P) and a biological control (BD Plasma Count, reference 338331). Absolute quantifications were performed by counting 10,000 of the standard beads included in the BD Trucount Tubes. The protoplast dilutions used for cytometry quantifications contained absolute protoplast numbers within the 5,000–10,000 range, which was close to the number of beads used as a standard. The number of protoplasts per μl was calculated based on the number of standard beads, and the number of protoplasts per mg of fresh weight was calculated based on the original fresh weight used to form the protoplasts (see above).

The reliability of the differences in the number of protoplasts formed was analysed by analysis of variance with Tukey's HSD *post hoc* analysis. Differences were considered significant if the *P* value was equal to or less than 0.05 (asterisks in Fig. 7a).

### Data availability

The authors declare that the data supporting the findings of this study are available within the article and its supplementary information files or from the corresponding authors on request.

## Additional information

**How to cite this article**: Yagüe, P. *et al*. Subcompartmentalization by cross-membranes during early growth of Streptomyces hyphae. *Nat. Commun.* 7:12467 doi: 10.1038/ncomms12467 (2016).

## Supplementary Material

Supplementary Information Supplementary Figures 1-6, and Supplementary Table 1

Supplementary Movie 1 Time-lapse analysis of FtsZ-eGFP expression and cellular localization in *S.* coelicolor grown on GYM over 15 hours.

## Figures and Tables

**Figure 1:**
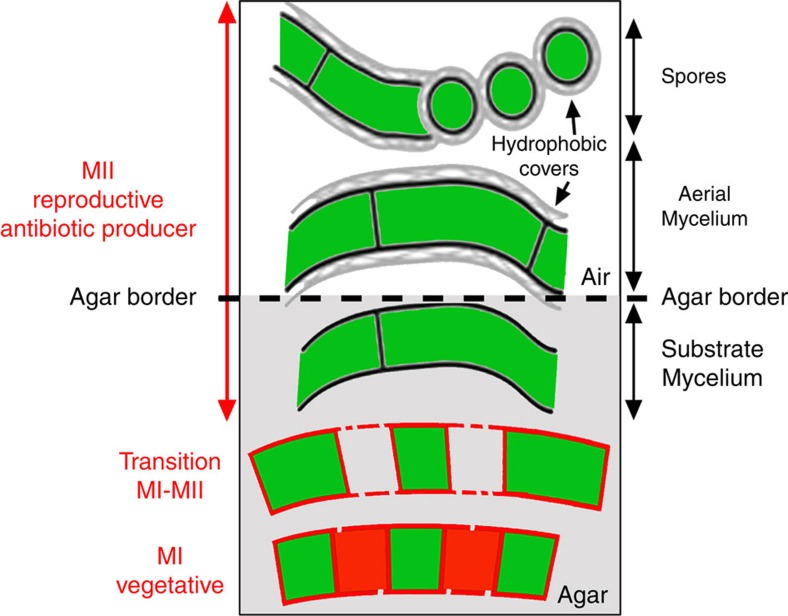
Development of *Streptomyces* on solid agar plates. The *Streptomyces* developmental cycle^3^,^4^,^5^,^6^,^7^,^8^ along the transverse axis of the plate is illustrated (the agar border is indicated with a dashed line). Discontinuities in hyphal membranes represent changes in membrane permeability in dying cells. Red and green colours represent PI fluorescence (dying cells) and SYTO9 fluorescence (live cells), respectively. The traditional nomenclature for substrate and aerial mycelia is indicated in black letters (right); the new nomenclature for MI and MII stages is indicated in red letters (left).
