# Supplementary material for: Subcompartmentalization by cross-membranes during early growth of Streptomyces hyphae
Source: Nat Commun. 2016 Aug 12;7:12467. doi: 10.1038/ncomms12467 (PMC4990651; doi:10.1038/ncomms12467)

A

MI(12h) SYTO9-PI staining

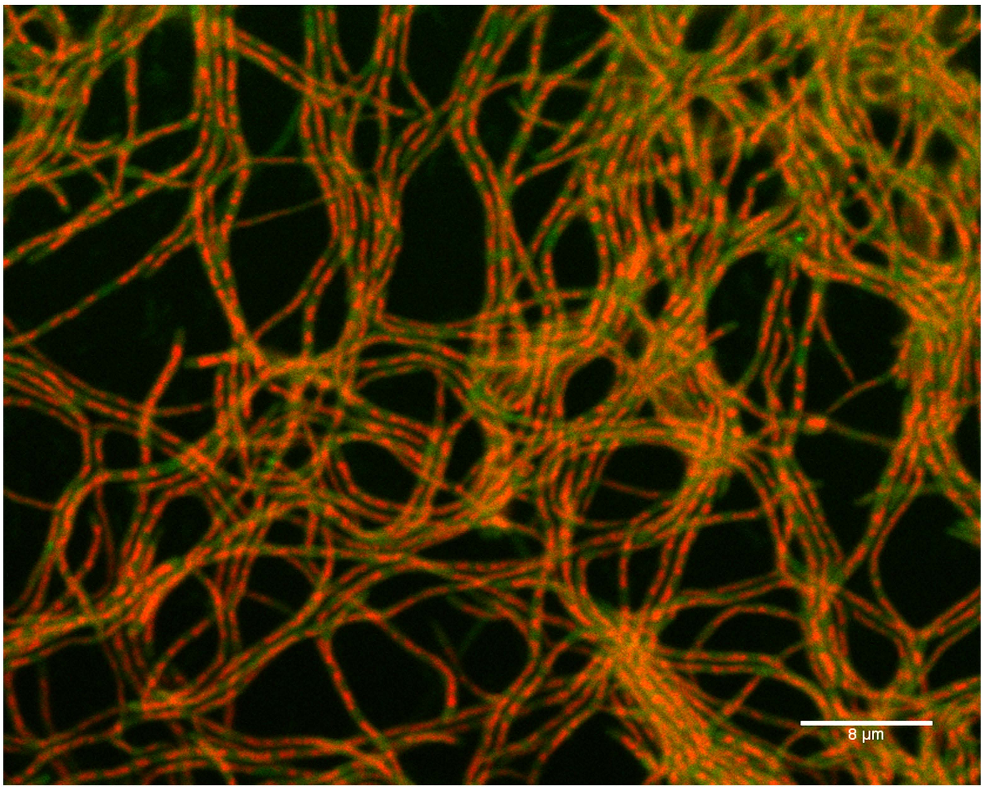

B

MI(12h) Contrast mode

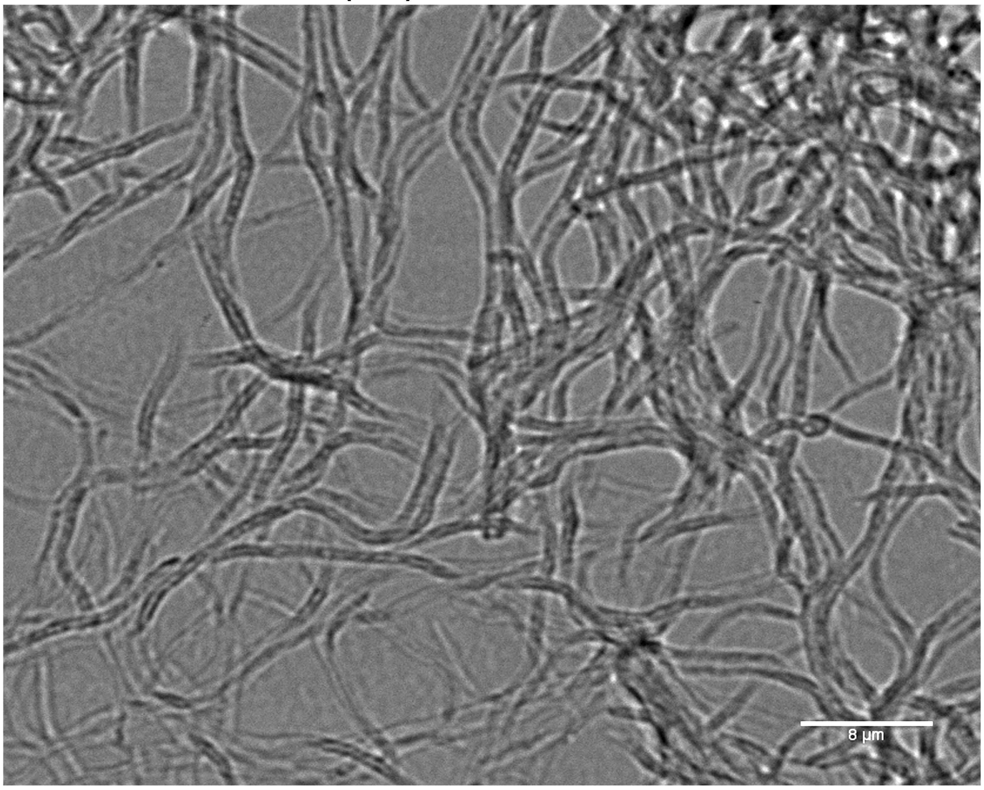

Supplementary Fig. 1. Images showing *S. coelicolor* hyphae compartmentalization. (A-D; G,H) MI; (E) transition from MI to MII;(F) sporulating hyphae. Segments and Z-rings used to quantify compartment lengths and Z-ring spacing are marked. Scale bars correspond to 8 µm.

(A) MI hyphae stained with PI (red) and SYTO9 (green).

(B) Contrast mode of the image shown in (A).

C

MI(10h) SYTO9-PI staining

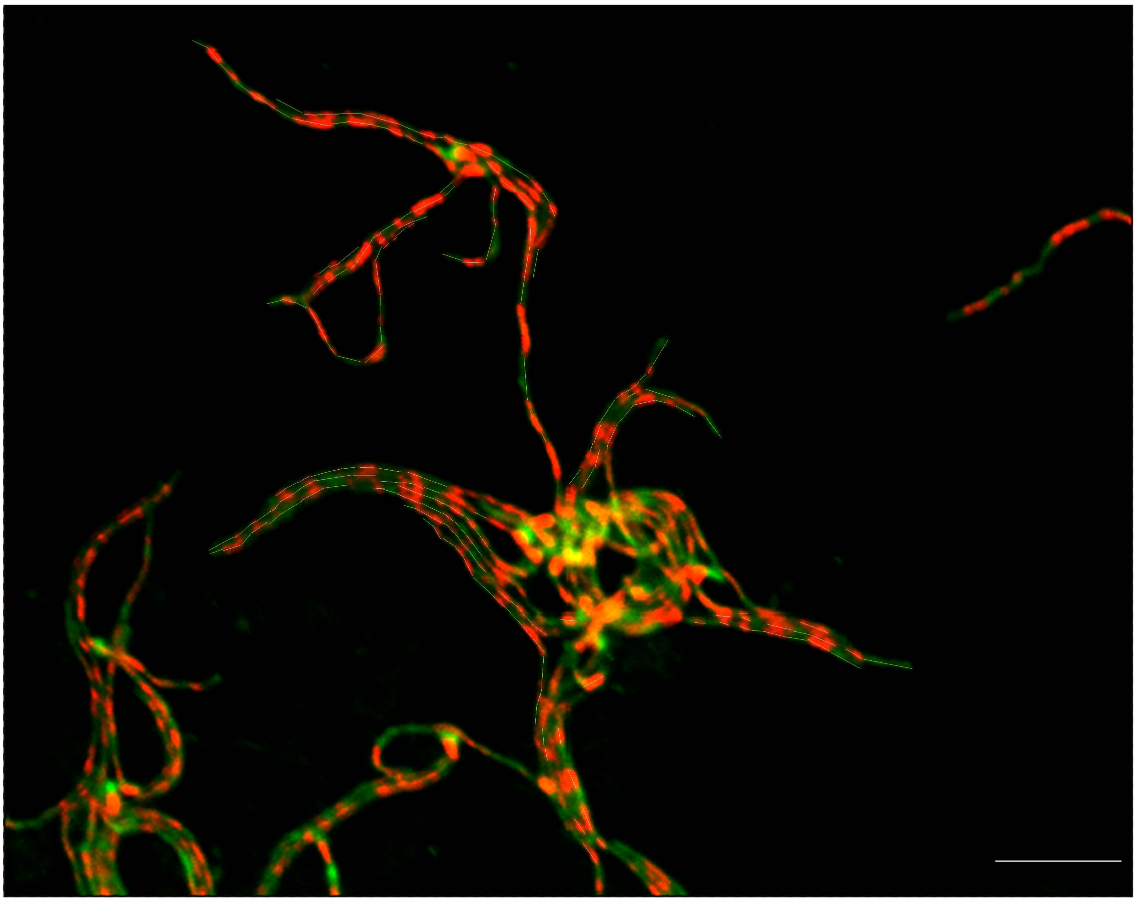

PI stained segments
- 103 segments
- Mean: 1.12 µm
- SD 0.38 µm
- Median 1.05 µm

SYTO9 stained segments
- 103 segments
- Mean: 0.92 µm
- SD 0.25 µm
- Median 0.88 µm

Supplementary Fig. 1. Images showing *S. coelicolor* hyphae compartmentalization. (A-D; G,H) MI; (E) transition from MI to MII;(F) sporulating hyphae. Segments and Z-rings used to quantify compartment lengths and Z-ring spacing are marked. Scale bars correspond to 8 µm.

(C) Master image used to quantify PI- and SYTO9-stained segments in the MI hyphae.

D

MI(10h) YOPRO-1 PI staining

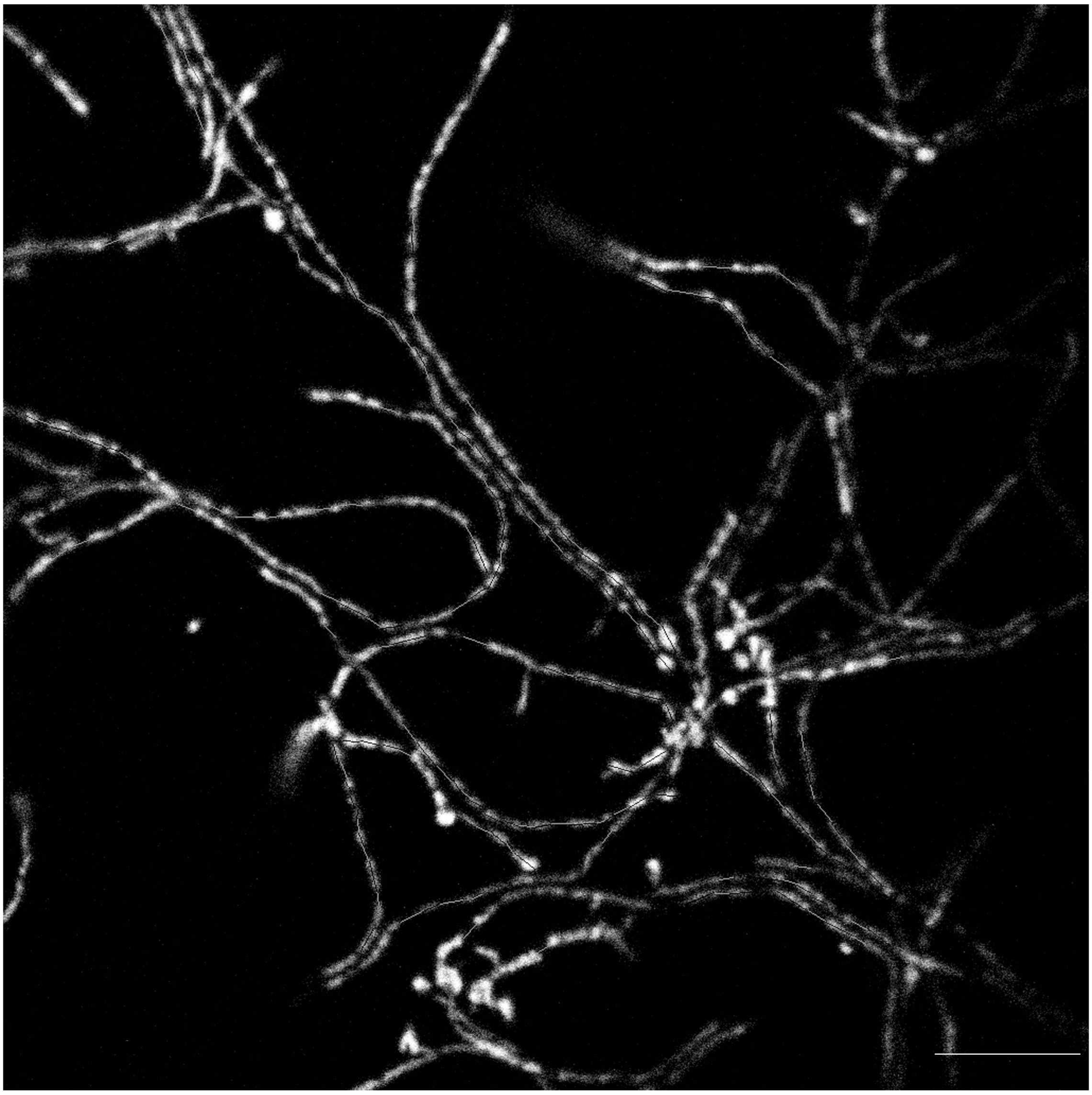

YOPRO-1/PI stained segments
- 102 segments
- Mean: 1.1 µm
- SD 0.41 µm
- Median 0.99 µm

Unstained segments
- 100 segments
- Mean: 0.94 µm
- SD 0.21 µm
- Median 0.91 µm

Supplementary Fig. 1. Images showing *S. coelicolor* hyphae compartmentalization. (A-D; G,H) MI; (E) transition from MI to MII;(F) sporulating hyphae. Segments and Z-rings used to quantify compartment lengths and Z-ring spacing are marked. Scale bars correspond to 8 µm.

(D) Master image used to quantify YOPRO-1- and PI-stained and unstained segments in the MI hyphae. The same segments were stained with YOPRO-1 and PI.

E

Transition MI-MII(18h) SYTO9-PI staining

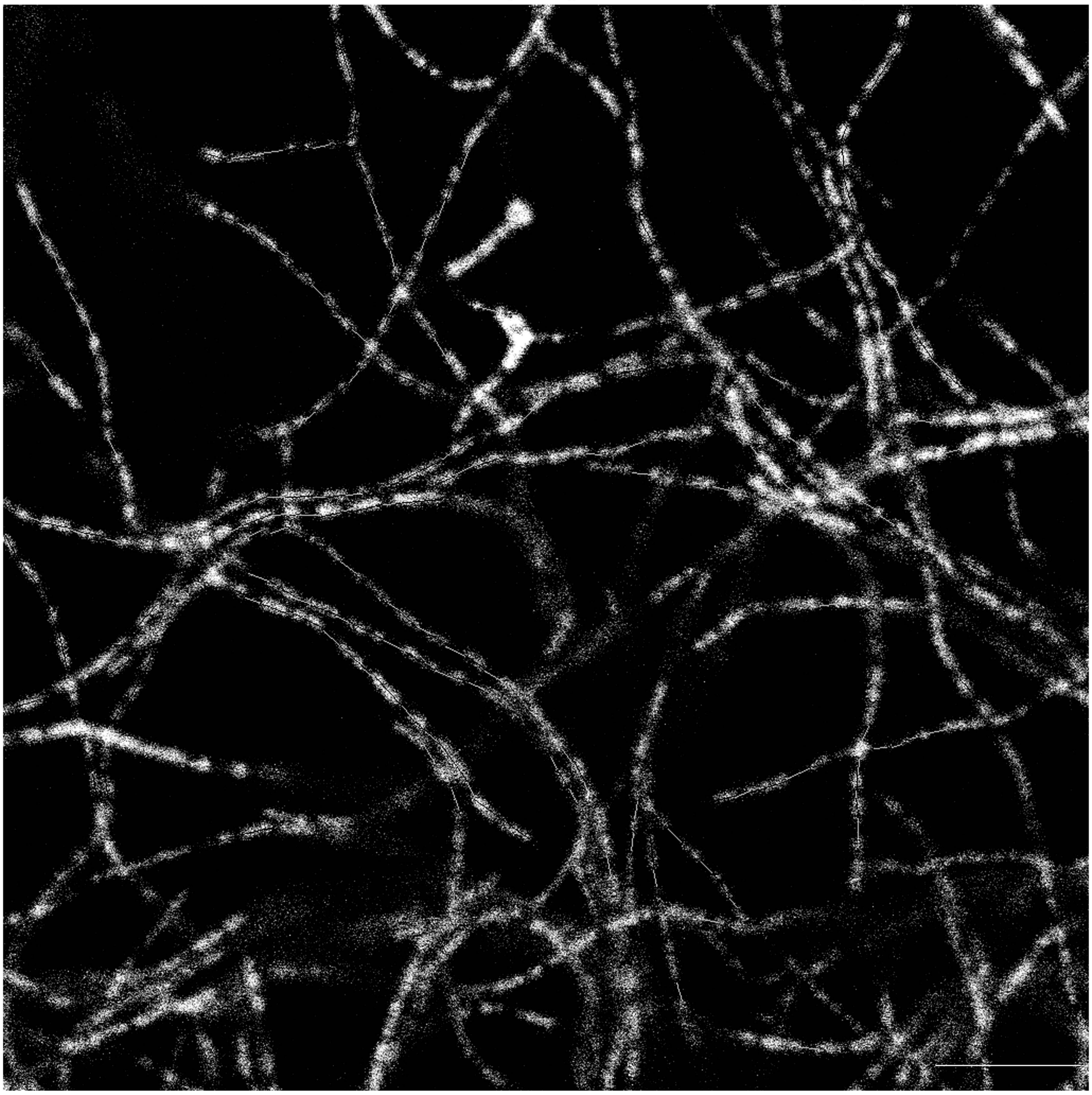

SYTO9 stained segments
- 102 segments
- Mean: 1.5 μm
- SD 0.55 μm
- Median 1.51 μm

Unstained segments
- 101 segments
- Mean: 0.91 μm
- SD 0.17 μm
- Median 0.93 μm

Supplementary Fig. 1. Images showing *S. coelicolor* hyphae compartmentalization. (A-D; G,H) MI; (E) transition from MI to MII;(F) sporulating hyphae. Segments and Z-rings used to quantify compartment lengths and Z-ring spacing are marked. Scale bars correspond to 8 μm.

(E) Master image used to quantify SYTO9-stained and unstained cellular segments in the transition from the MI to MII stage. Cells were stained with SYTO9 and PI, but at this stage, no cells were labelled by PI (see the manuscript for details).

F

Spores(72h) SYTO9-PI staining

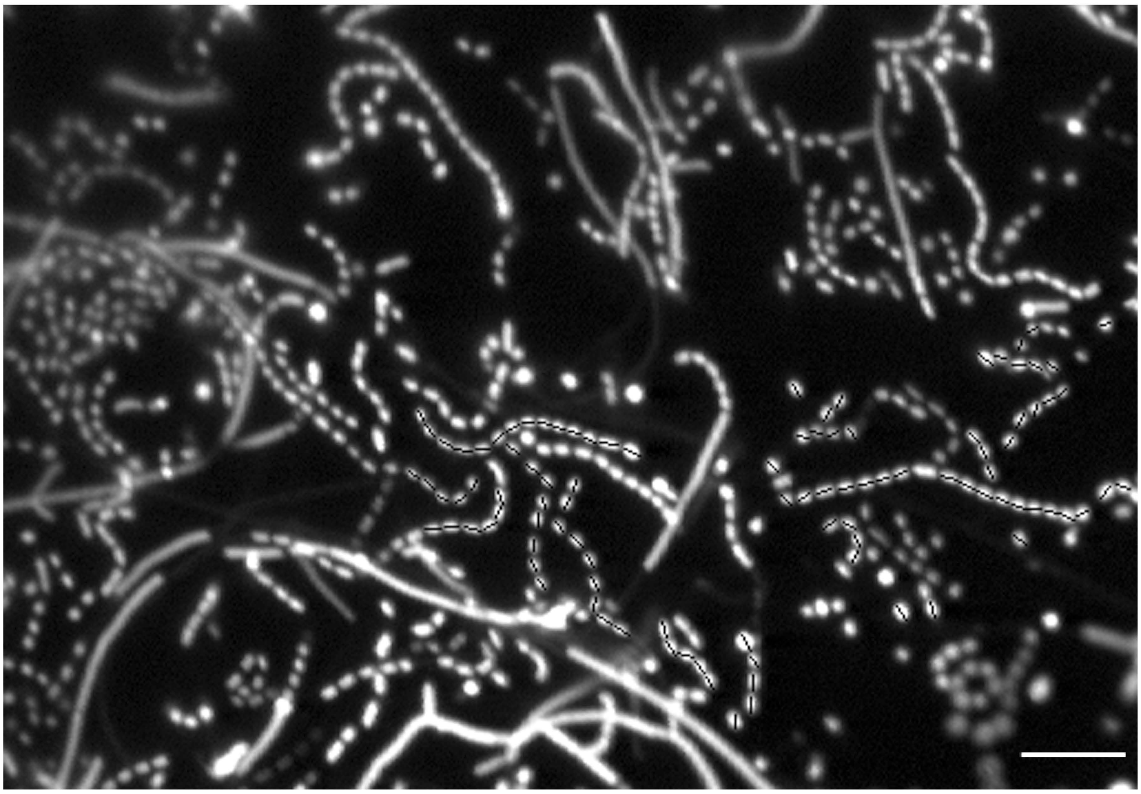

Spore diameter

- 101 segments
- Mean: 0.99 µm
- SD 0.16 µm
- Median 0.98 µm

Supplementary Fig. 1. Images showing *S. coelicolor* hyphae compartmentalization. (A-D; G,H) MI; (E) transition from MI to MII;(F) sporulating hyphae. Segments and Z-rings used to quantify compartment lengths and Z-ring spacing are marked. Scale bars correspond to 8 µm.

(F) Master image used to quantify spore diameter. Hyphae were stained with PI and SYTO9.

G eGFP-FtsZ (maximum projection of the time lapse experiment)

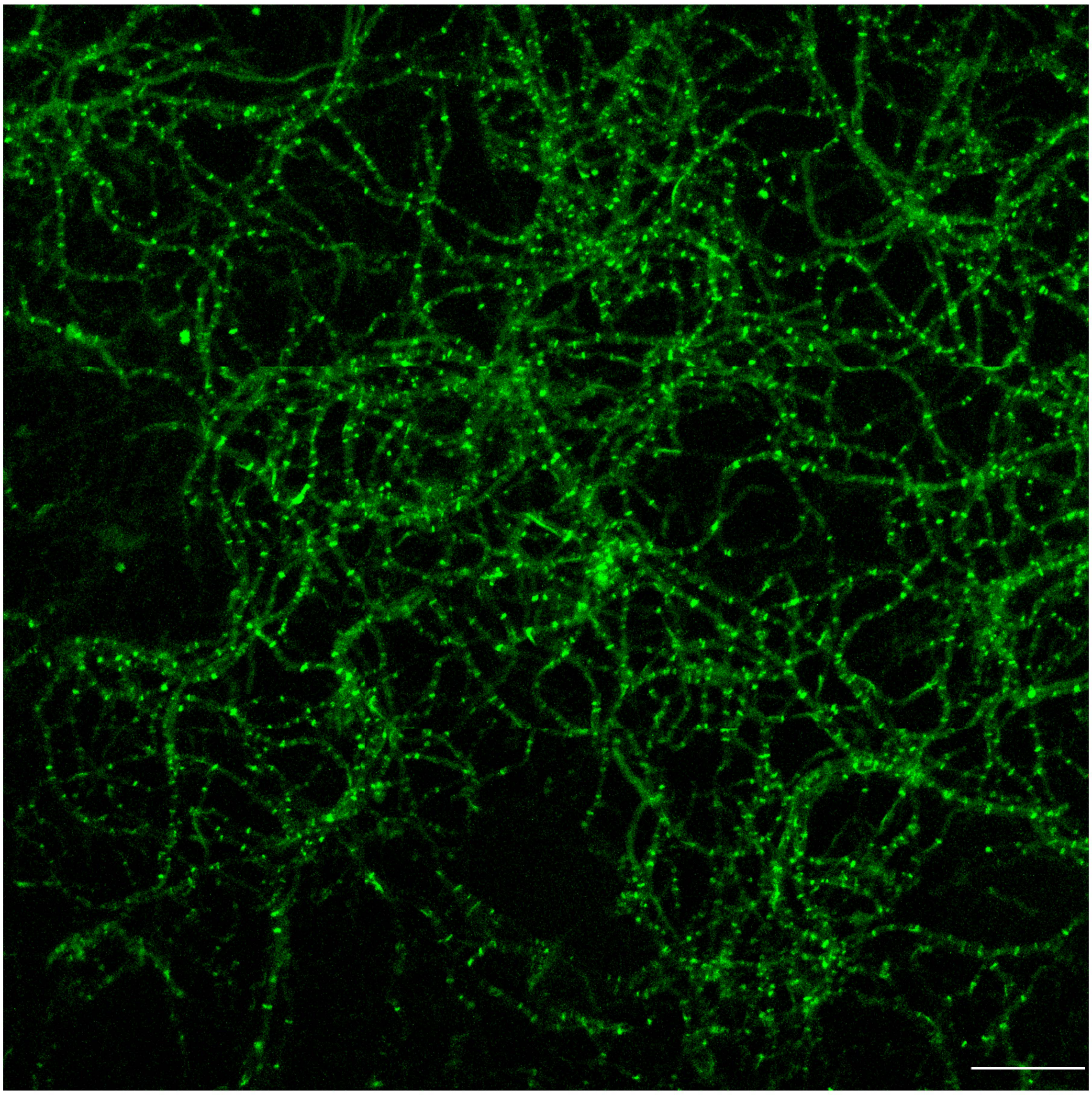

Supplementary Fig. 1. Images showing *S. coelicolor* hyphae compartmentalization. (A-D; G,H) MI; (E) transition from MI to MII;(F) sporulating hyphae. Segments and Z-rings used to quantify compartment lengths and Z-ring spacing are marked. Scale bars correspond to 8 μm.

(G) Master image (maximum projection of the time lapse experiment) used to quantify Z-ring spacing in *S. coelicolor* FM145 expressing FtsZ-eGFP.

H eGFP-FtsZ (maximum projection) processed for quantification

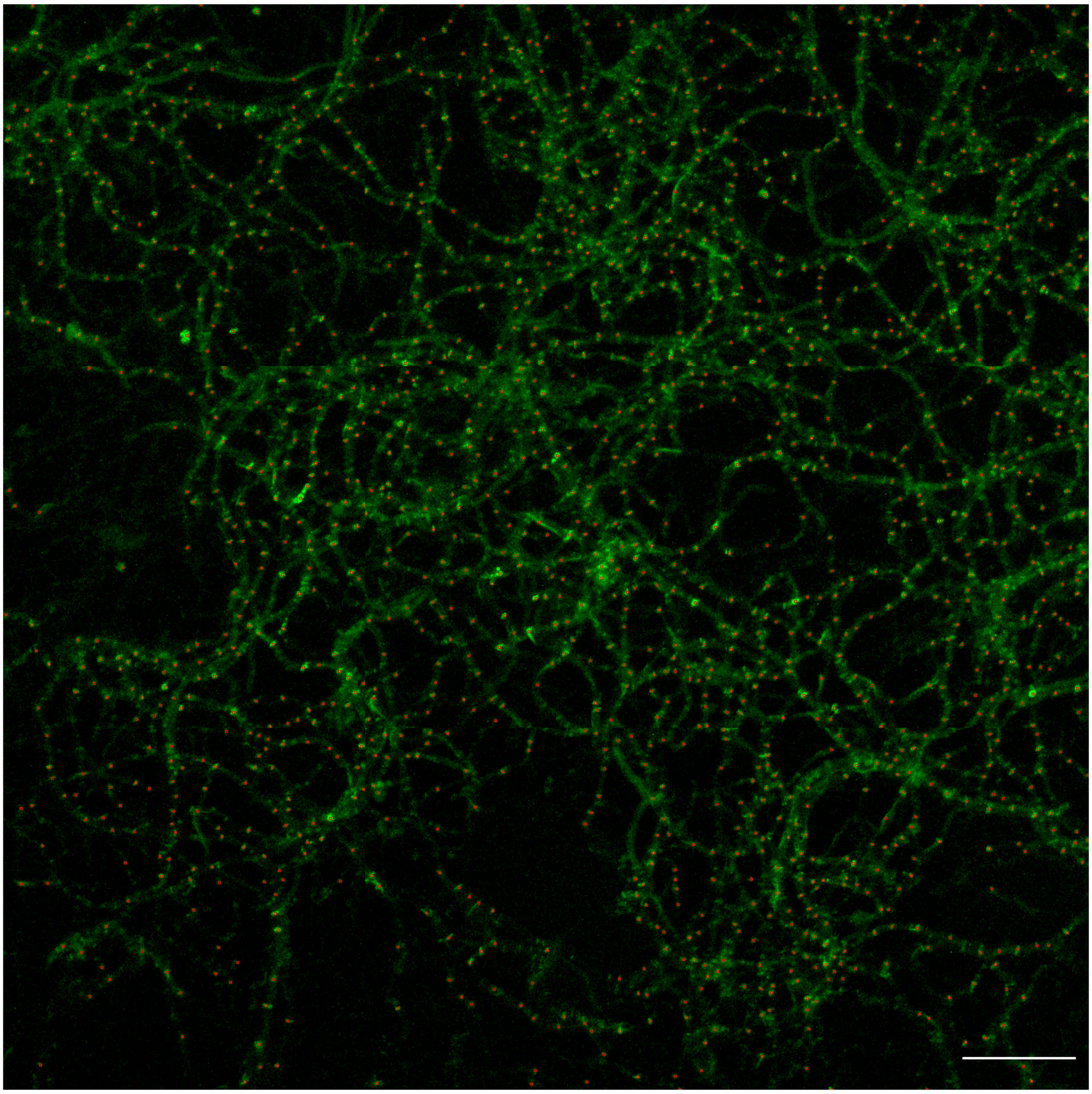

eGFP Z-ring spacing

- 1514 Z-rings
- Mean: 1.12 µm
- SD 0.48 µm
- Median 1.03 µm

Supplementary Fig. 1. Images showing *S. coelicolor* hyphae compartmentalization. (A-D; G,H) MI; (E) transition from MI to MII;(F) sporulating hyphae. Segments and Z-rings used to quantify compartment lengths and Z-ring spacing are marked. Scale bars correspond to 8 µm.

(H) Same image as (G) processed for quantification.

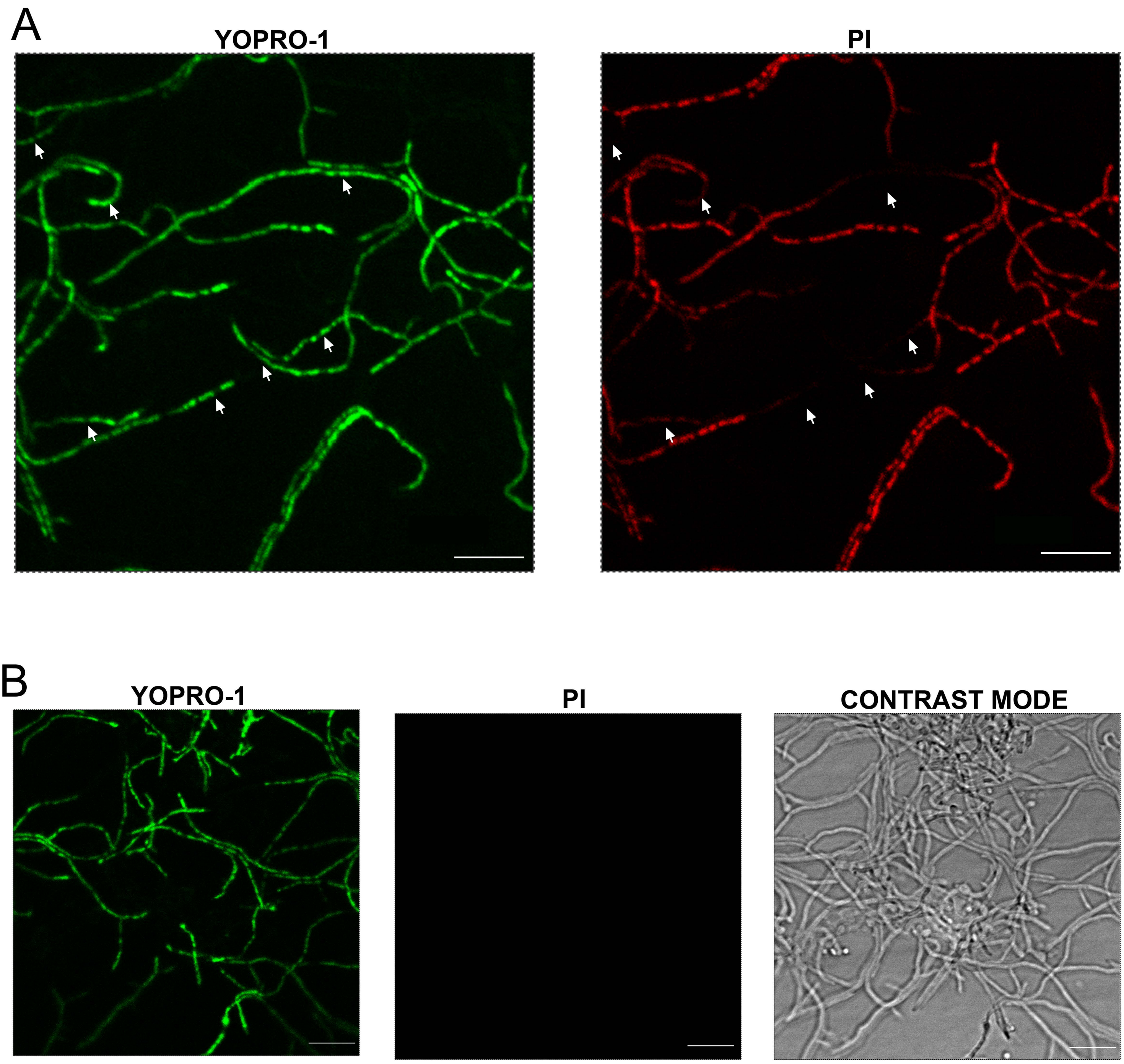

Supplementary Fig. 2. Permeability of MI hyphae (10-hours) to YOPRO-1 and PI. Scale bars correspond to 8 μm.

(A) Slowly processed samples (minutes). All segments stained with PI (red) are also stained with YOPRO-1 (green), but some segments stained with YOPRO-1 are not stained with PI (labelled with arrows).

(B) Rapidly processed samples (seconds). Dying cells are permeable to YOPRO-1 but not to PI.

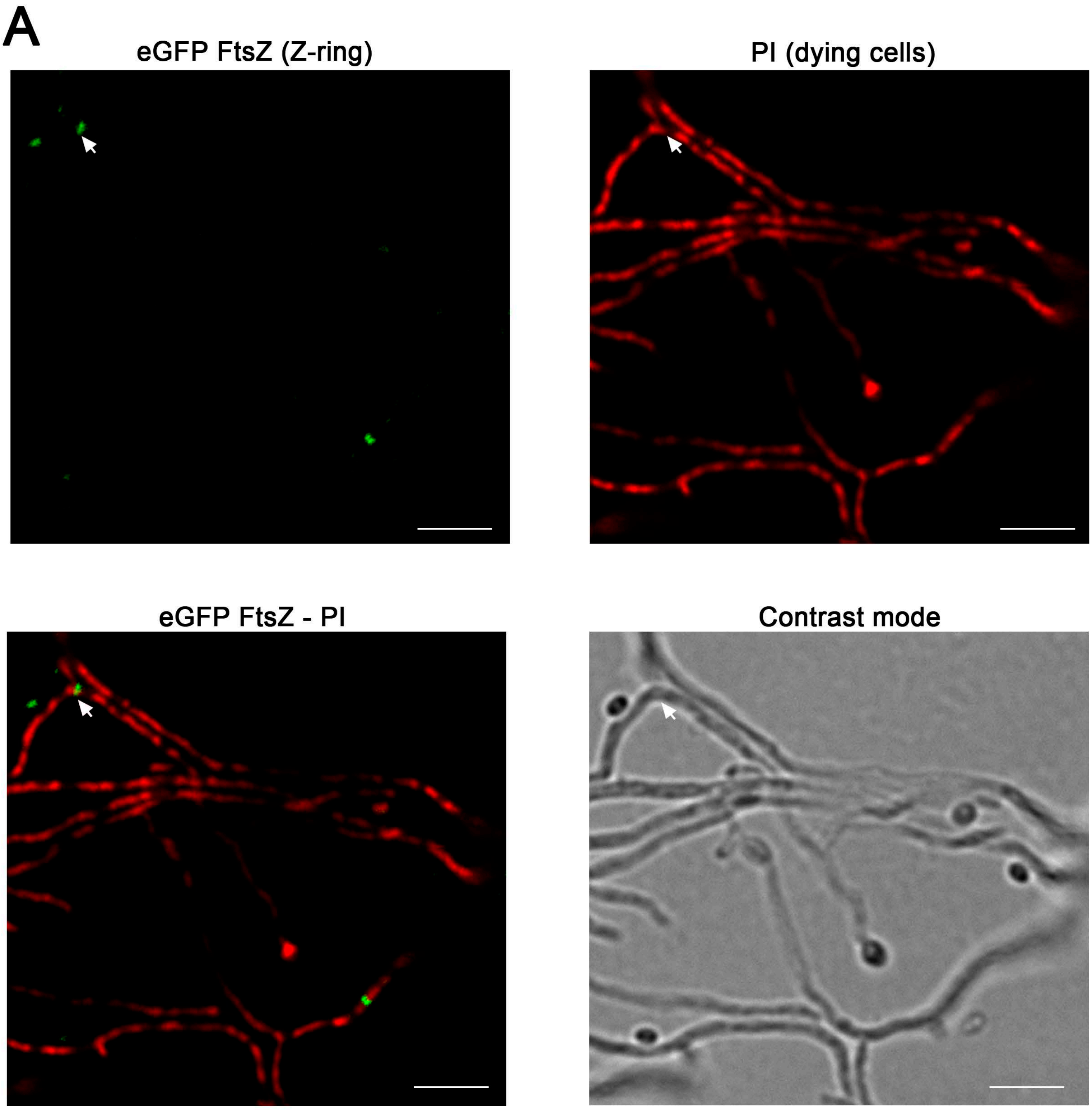

Supplementary Fig. 3. Images showing the colocalization of Z-rings, PI permeability barriers, cross-membranes and cross-walls. Scale bars correspond to 4 µm. (A-D) eGFP-FtsZ (Z-rings, green) and PI (dying cells, red) staining. Arrows indicate Z-rings colocalizing with PI permeability borders. Ten-hour GYM solid cultures.

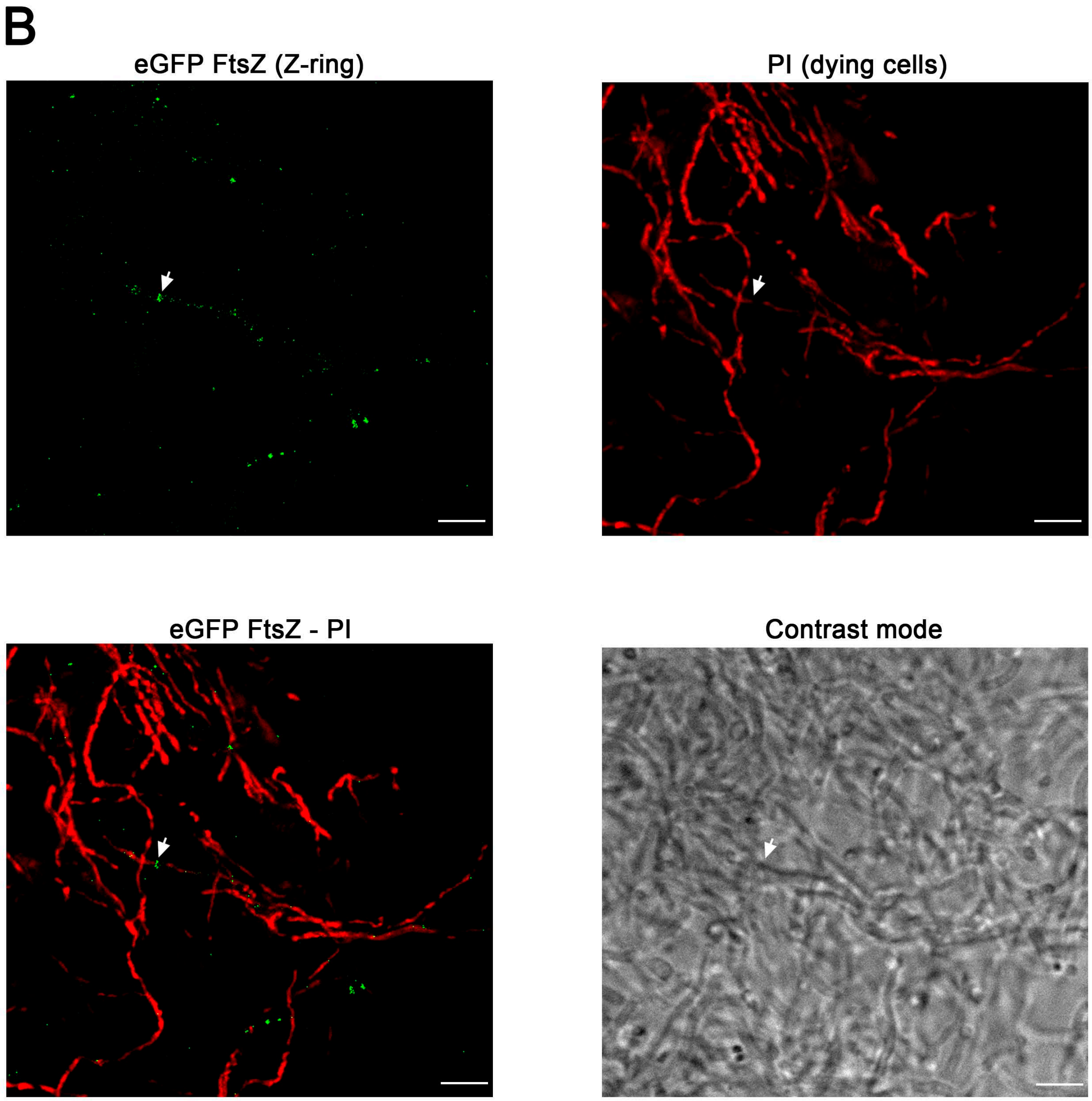

Supplementary Fig. 3. Images showing the colocalization of Z-rings, PI permeability barriers, cross-membranes and cross-walls. Scale bars correspond to 4 µm.
(A-D) eGFP-FtsZ (Z-rings, green) and PI (dying cells, red) staining. Arrows indicate Z-rings colocalizing with PI permeability borders. Ten-hour GYM solid cultures.

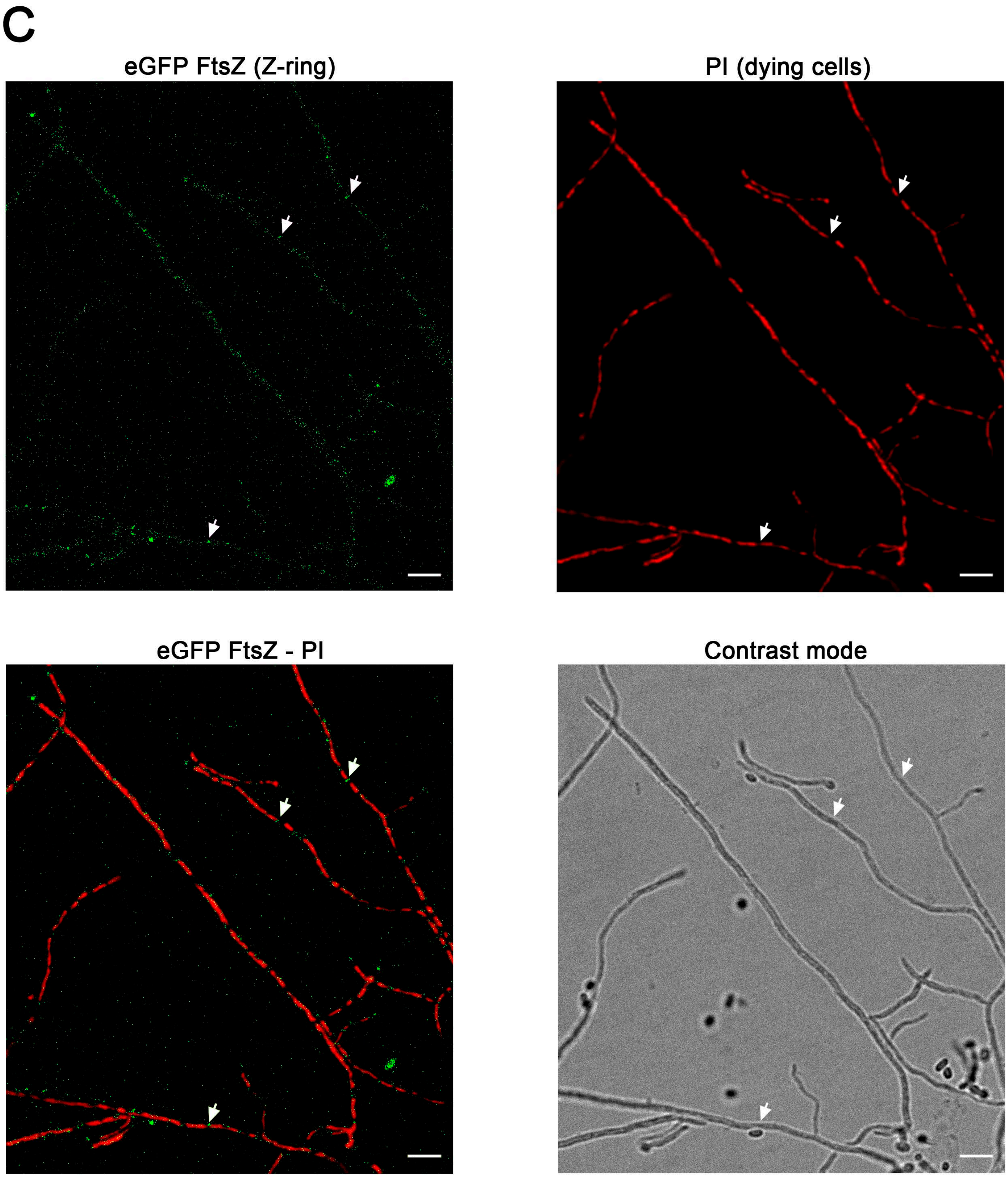

Supplementary Fig. 3. Images showing the colocalization of Z-rings, PI permeability barriers, cross-membranes and cross-walls. Scale bars correspond to 4 μm.
(A-D) eGFP-FtsZ (Z-rings, green) and PI (dying cells, red) staining. Arrows indicate Z-rings colocalizing with PI permeability borders. Ten-hour GYM solid cultures.

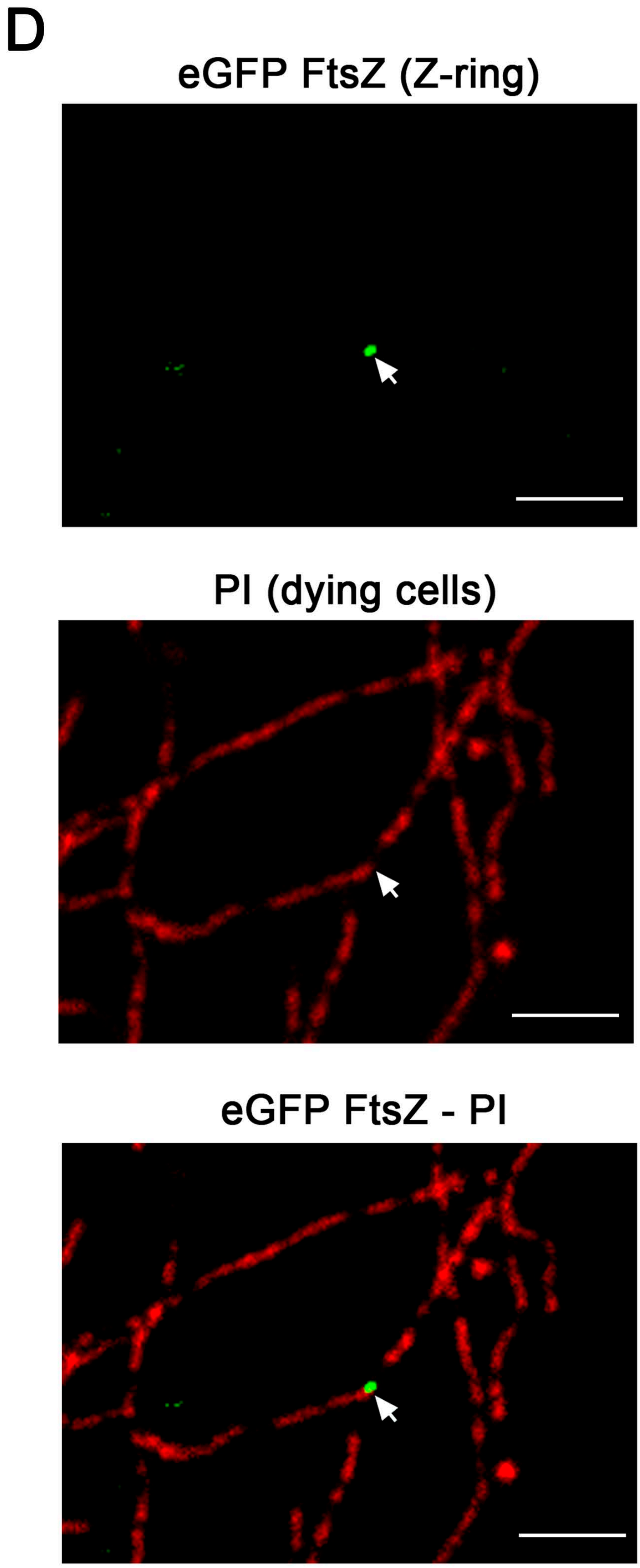

Supplementary Fig. 3. Images showing the colocalization of Z-rings, PI permeability barriers, cross-membranes and cross-walls. Scale bars correspond to 4 µm.
(A-D) eGFP-FtsZ (Z-rings, green) and PI (dying cells, red) staining. Arrows indicate Z-rings colocalizing with PI permeability borders. Ten-hour GYM solid cultures.

E

HADA(blue) and PI (red)

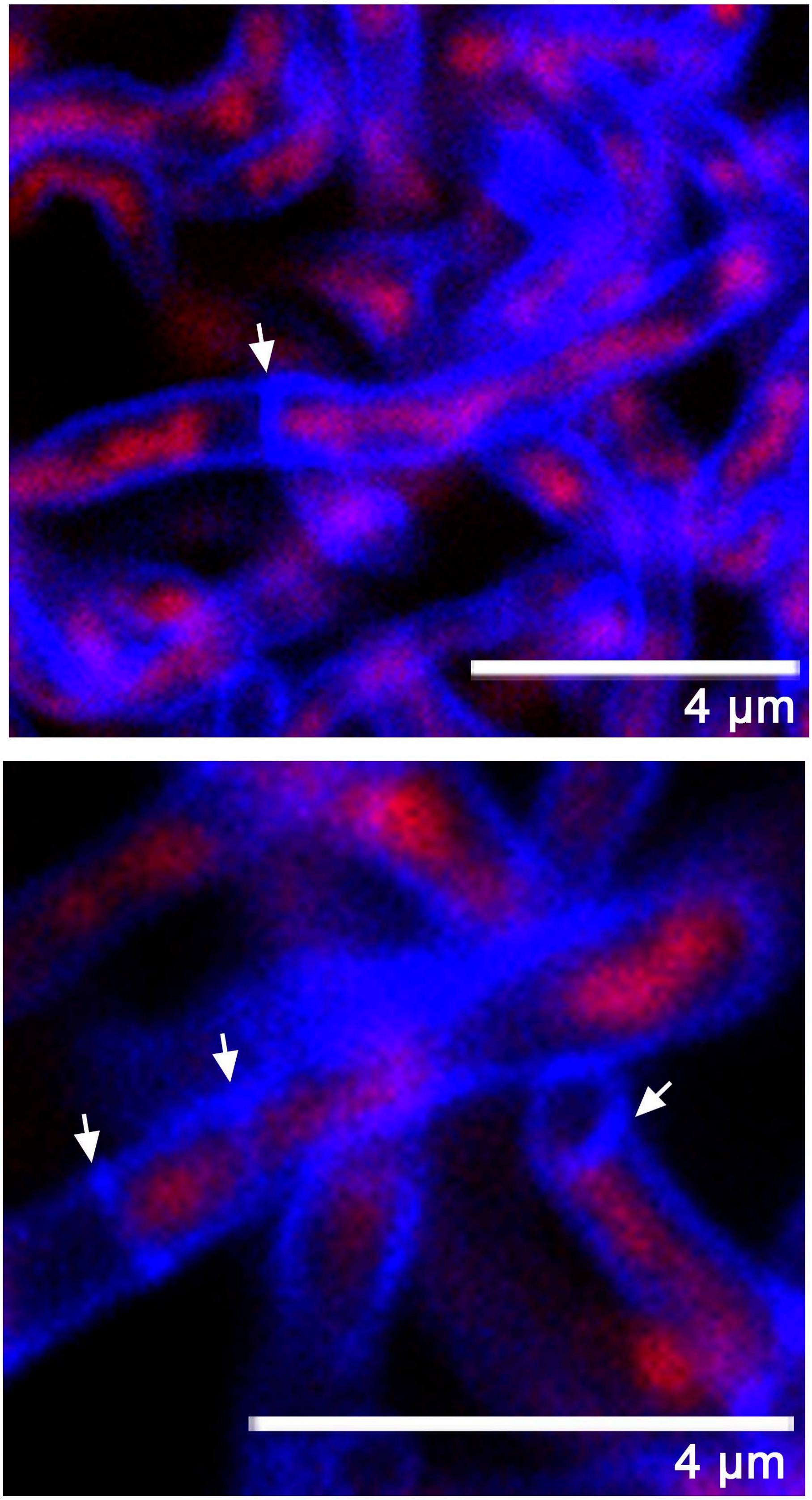

Supplementary Fig. 3. Images showing the colocalization of Z-rings, PI permeability barriers, cross-membranes and cross-walls. Scale bars correspond to 4 µm.

(E) HADA (cell wall, blue) and PI (dying cells, red) staining. Arrows indicate the colocalization between cell walls and PI permeability barriers. Fifteen-hour liquid GYM cultures.

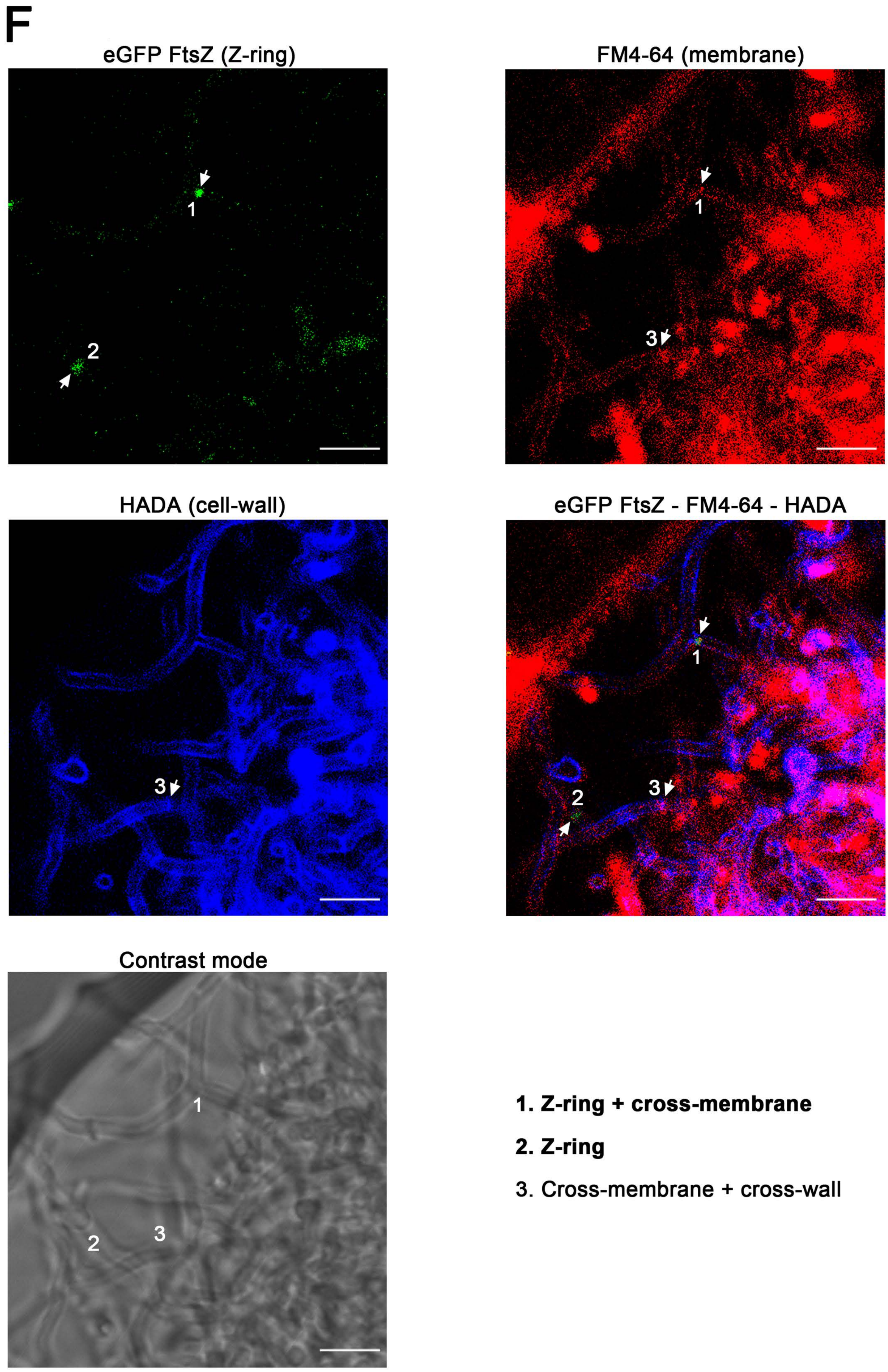

Supplementary Fig. 3. Images showing the colocalization of Z-rings, PI permeability barriers, cross-membranes and cross-walls. Scale bars correspond to 4 µm.
(F-I) eGFP-FtsZ (Z-rings, green), HADA (cell wall, blue) and FM4-64 (membrane, red) staining. Z-rings, cross-membranes and/or cross-walls colocalize in some cases (arrows).

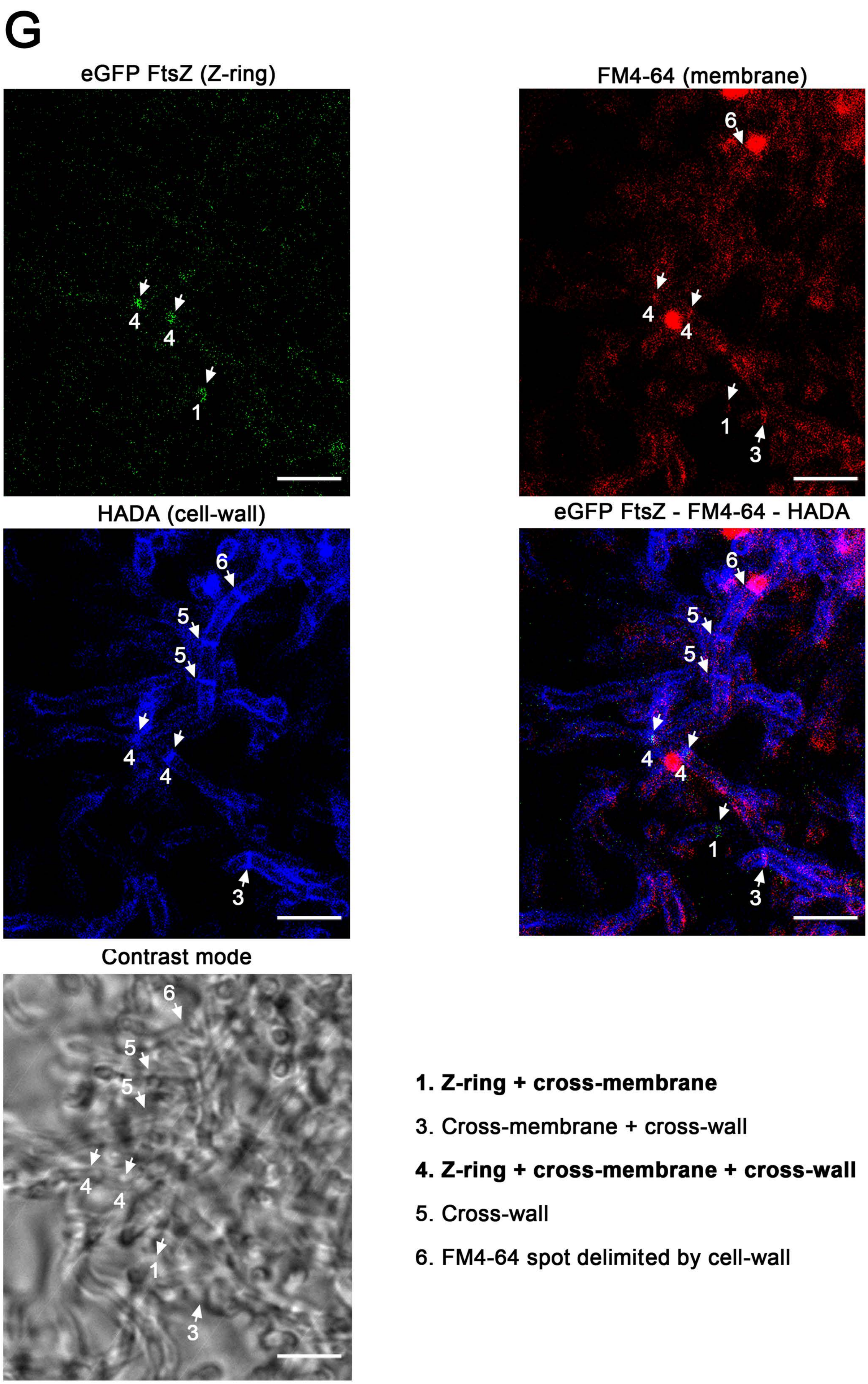

Supplementary Fig. 3. Images showing the colocalization of Z-rings, PI permeability barriers, cross-membranes and cross-walls. Scale bars correspond to 4 µm.
(F-I) eGFP-FtsZ (Z-rings, green), HADA (cell wall, blue) and FM4-64 (membrane, red) staining. Z-rings, cross-membranes and/or cross-walls colocalize in some cases (arrows).

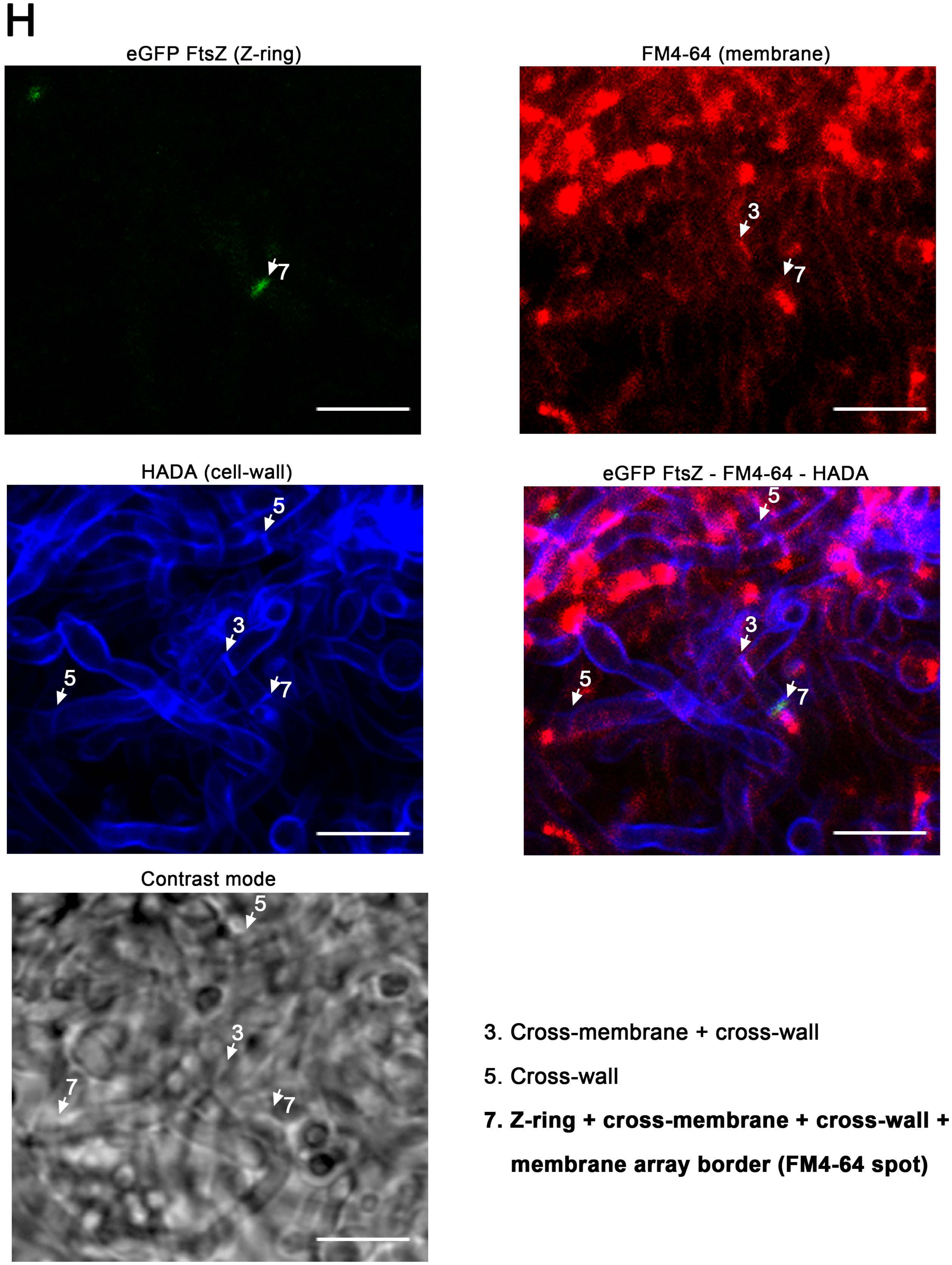

Supplementary Fig. 3. Images showing the colocalization of Z-rings, PI permeability barriers, cross-membranes and cross-walls. Scale bars correspond to 4 µm.
(F-I) eGFP-FtsZ (Z-rings, green), HADA (cell wall, blue) and FM4-64 (membrane, red) staining. Z-rings, cross-membranes and/or cross-walls colocalize in some cases (arrows).

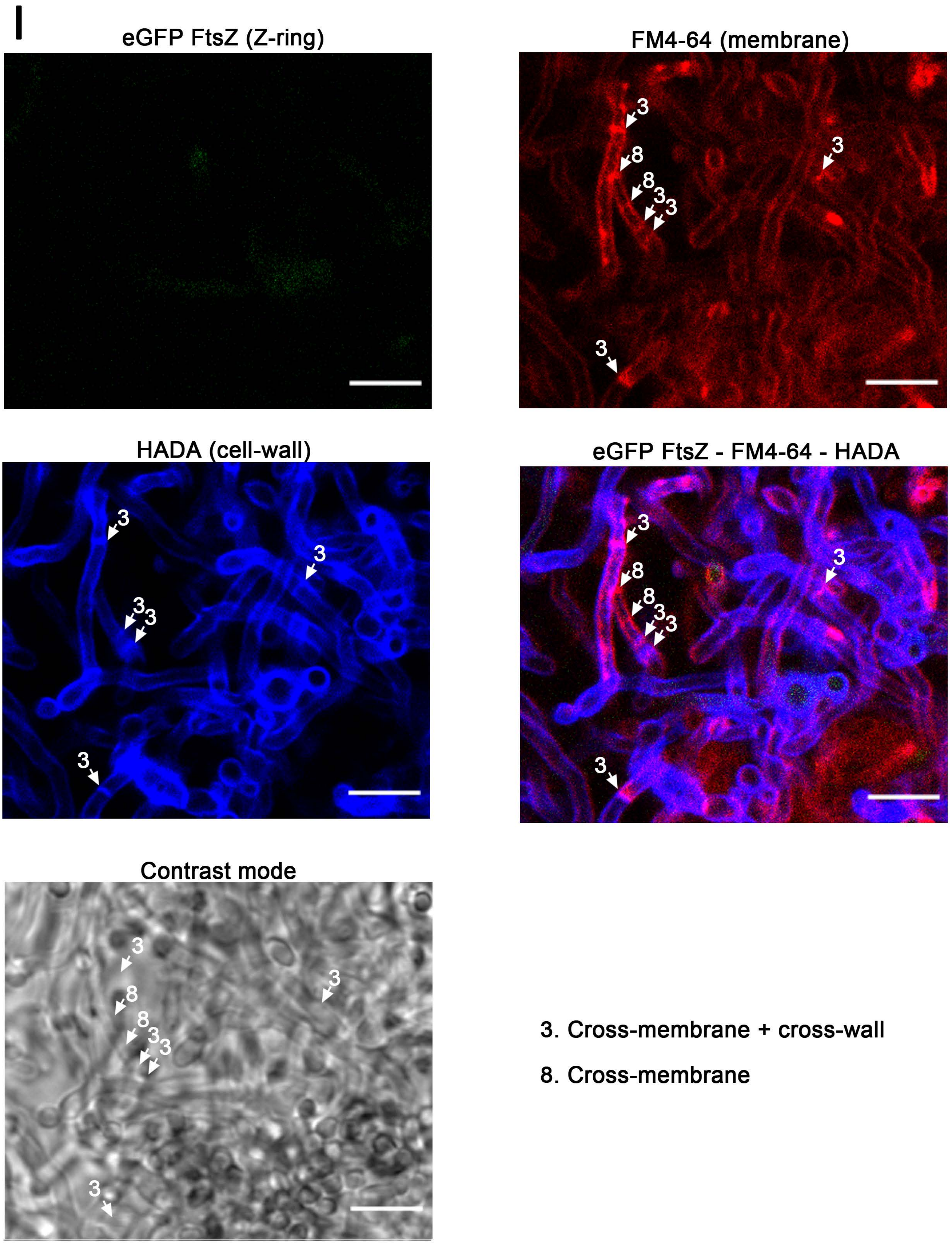

Supplementary Fig. 3. Images showing the colocalization of Z-rings, PI permeability barriers, cross-membranes and cross-walls. Scale bars correspond to 4 µm.
(F-I) eGFP-FtsZ (Z-rings, green), HADA (cell wall, blue) and FM4-64 (membrane, red) staining. Z-rings, cross-membranes and/or cross-walls colocalize in some cases (arrows).

A

SYTO9-PI staining (48h)

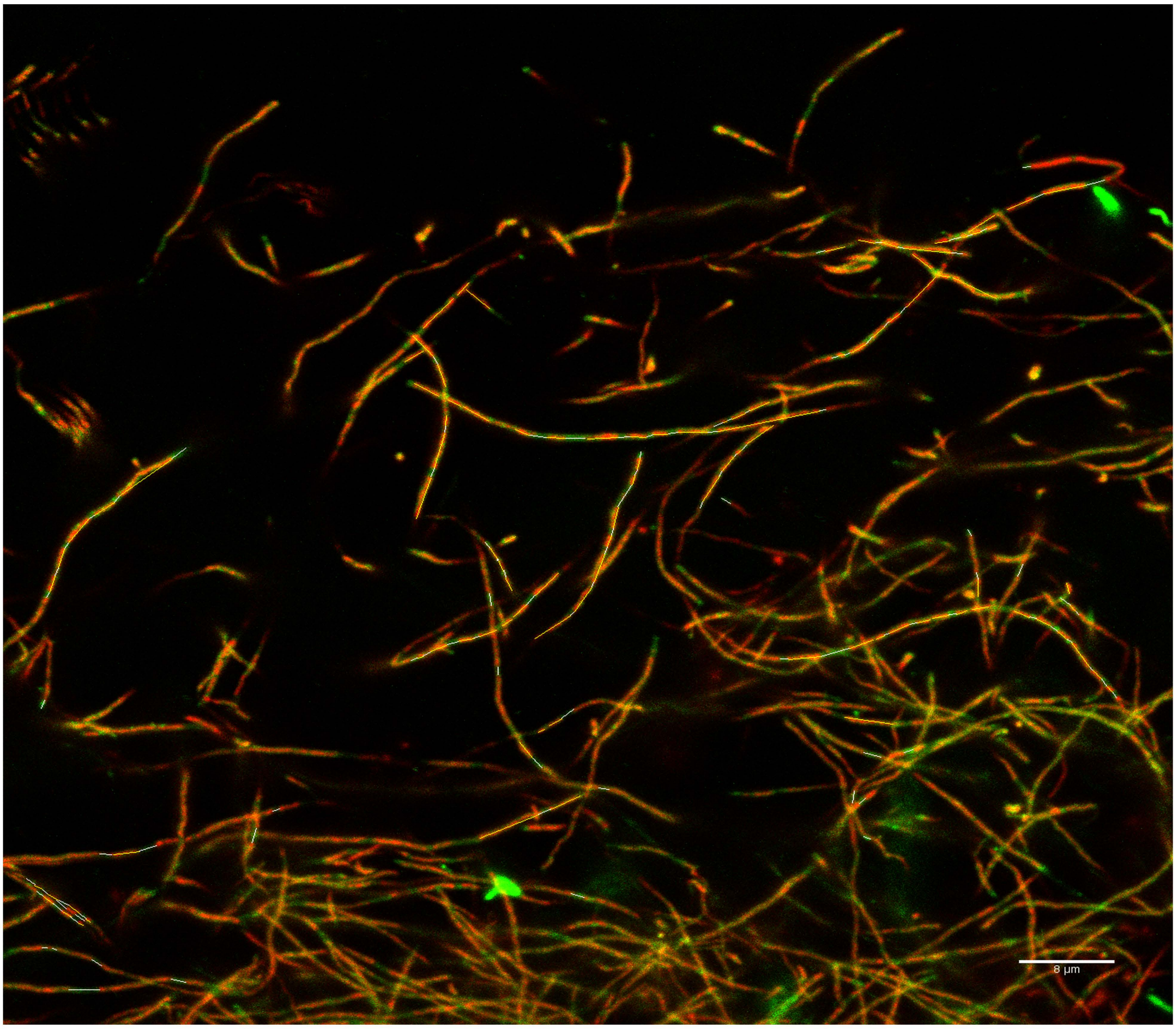

PI stained segments

- 100 segments
- Mean: 1.83 µm
- SD 1.27 µm
- Median 1.42 µm

SYTO9 stained segments

- 100 segments
- Mean: 0.85 µm
- SD 0.41 µm
- Median 0.74 µm

Supplementary Fig. 4. Master images used to quantify hyphal compartments in *ΔftsZ* HU133 (48 hours). Segments used for quantification of the compartment lengths are marked. Scale bars correspond to 8 µm.

(A) Master image used to quantify PI- (red) and SYTO9 (green)-stained segments.

B

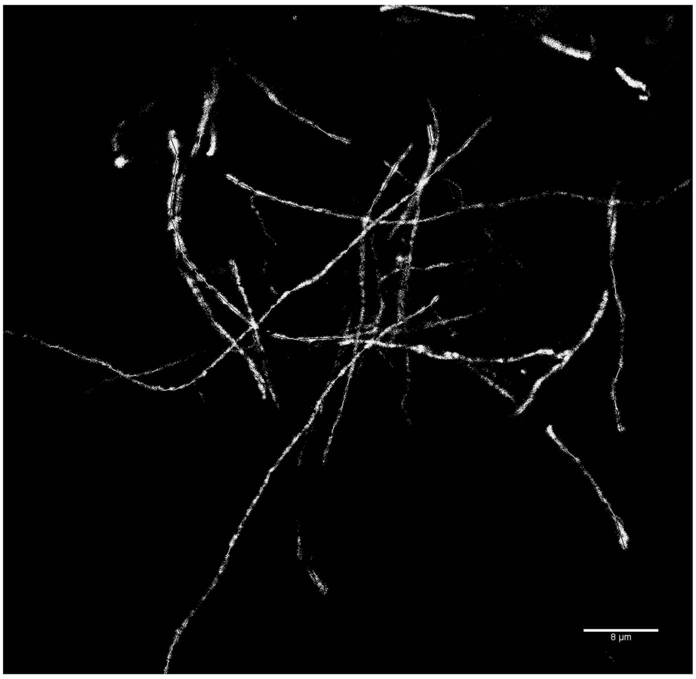

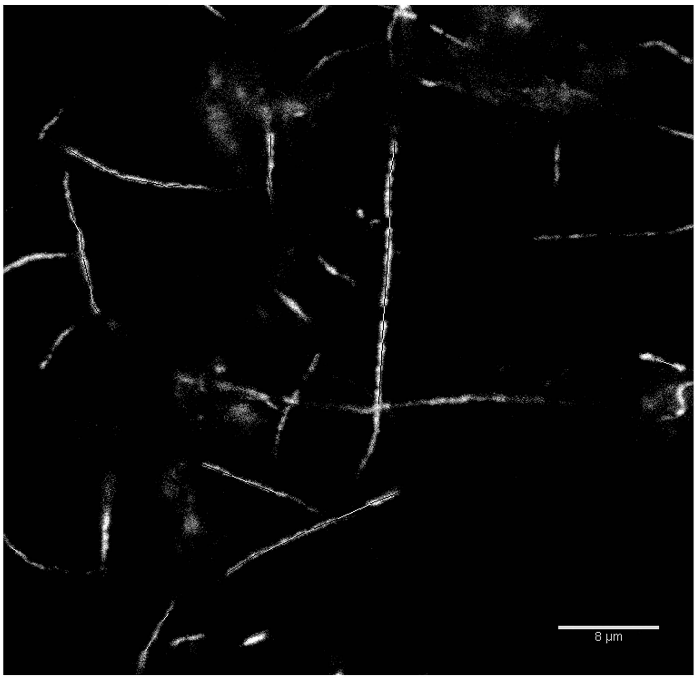

PI / YOPRO-1 stained segments
- 100 segments
- Mean: 1.83 µm
- SD 1.27 µm
- Median 1.42 µm

Unstained segments
- 102 segments
- Mean: 0.81 µm
- SD 0.36 µm
- Median 0.72 µm

Supplementary Fig. 4. Master images used to quantify hyphal compartments in *ΔftsZ* HU133 (48 hours). Segments used for quantification of the compartment lengths are marked. Scale bars correspond to 8 µm.

(B) Master image used to quantify YOPRO-1- and PI-stained and unstained segments. The same segments were stained with YOPRO-1 and PI.

A

HADA (fluorescent D-alanine) and contrast mode

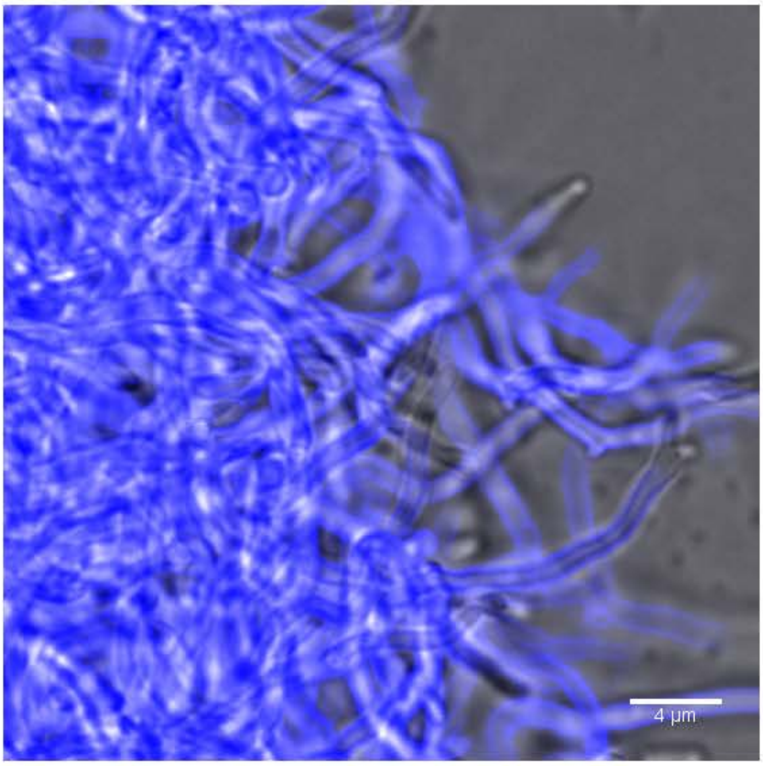

HADA (fluorescent D-alanine)

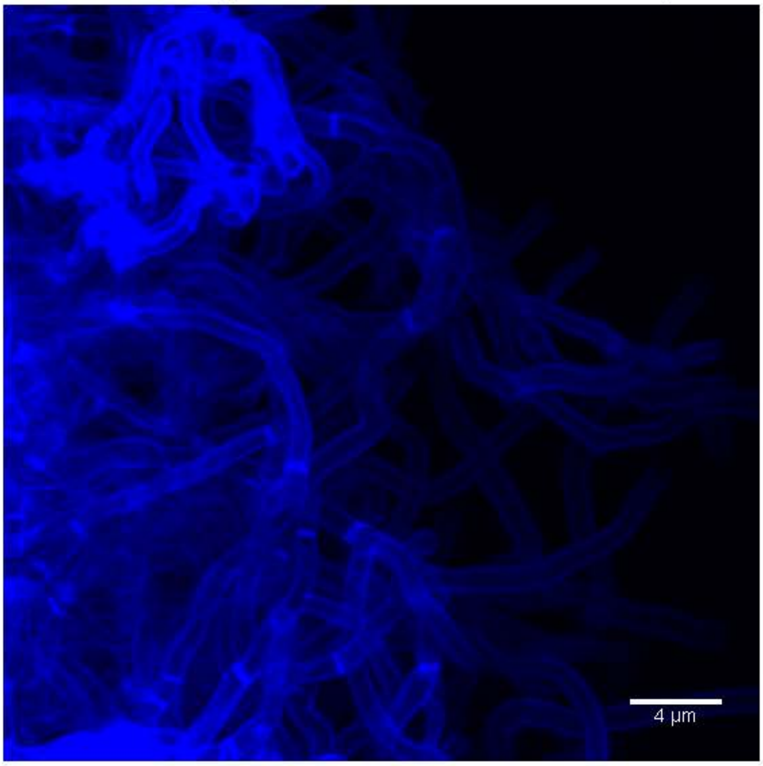

Supplementary Fig. 5. D-amino acid pulse-labeling staining (cell wall) of *S. coelicolor* and *ΔftsZ* HU133 (15 hours in liquid GYM medium). Scale bars correspond to 4 µm.
(A) *S. coelicolor*

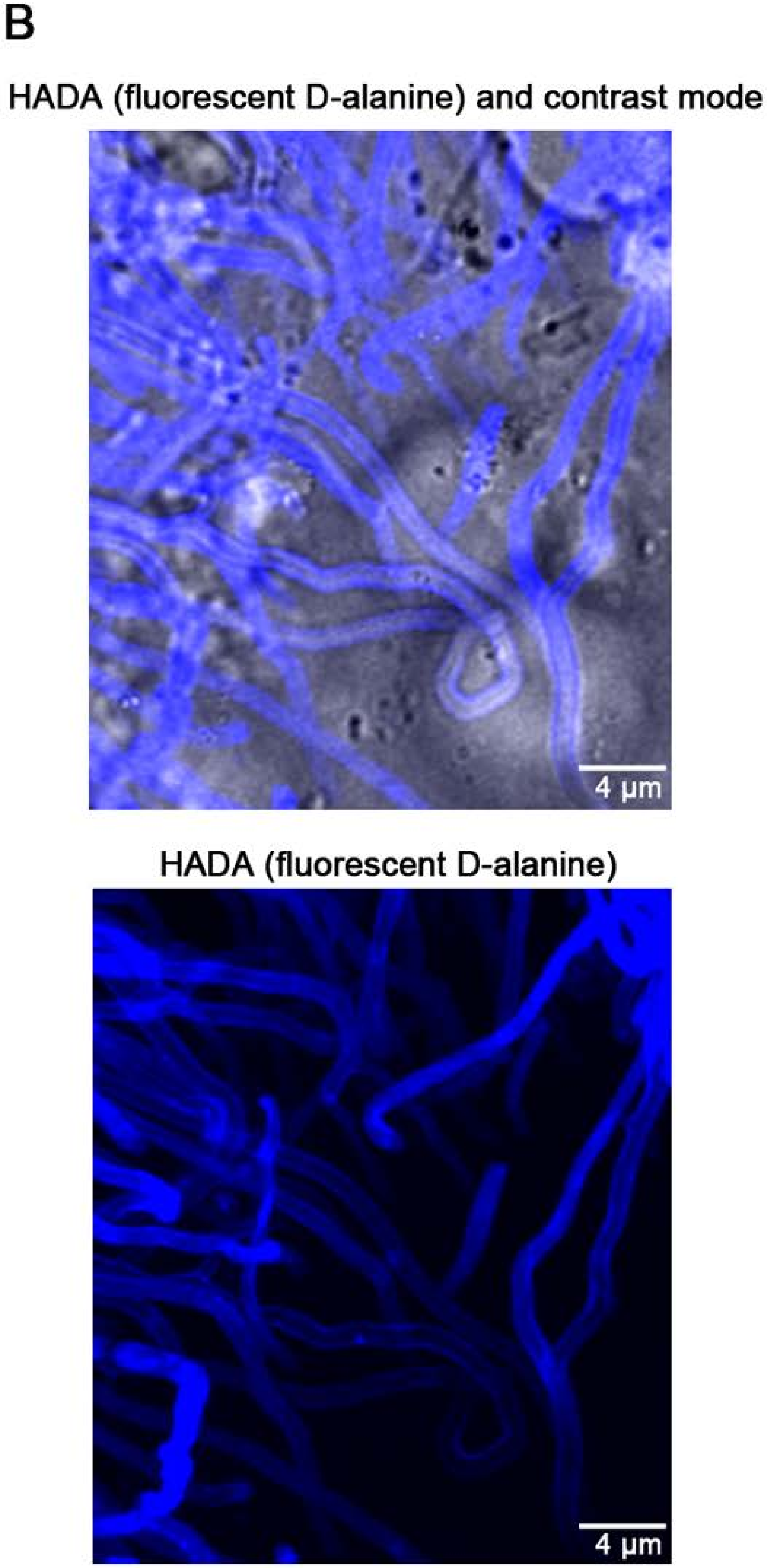

Supplementary Fig. 5. D-amino acid pulse-labeling staining (cell wall) of *S. coelicolor* and *ΔftsZ* HU133 (15 hours in liquid GYM medium). Scale bars correspond to 4 µm. (B) *ΔftsZ* HU133

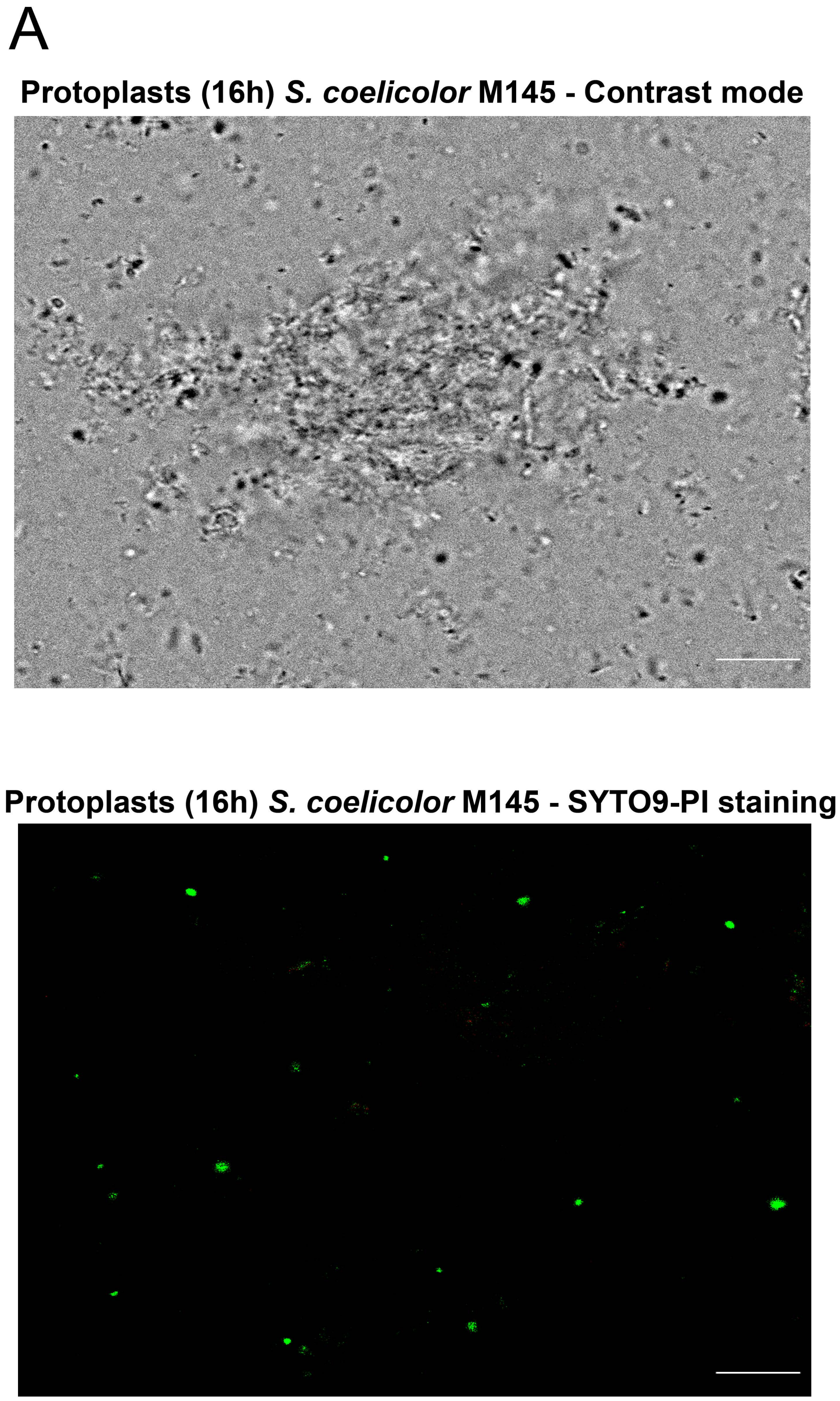

Supplementary Fig. 6. Protoplast formation and quantification. Scale bars correspond to 8 μm.

(A) Protoplasts stained with PI and SYTO9. Cell debris observed in phase contrast mode is devoid of stained cells, indicating a high efficiency of protoplast formation.

B

S. coelicolor M145 (16h) SYTO9-PI staining

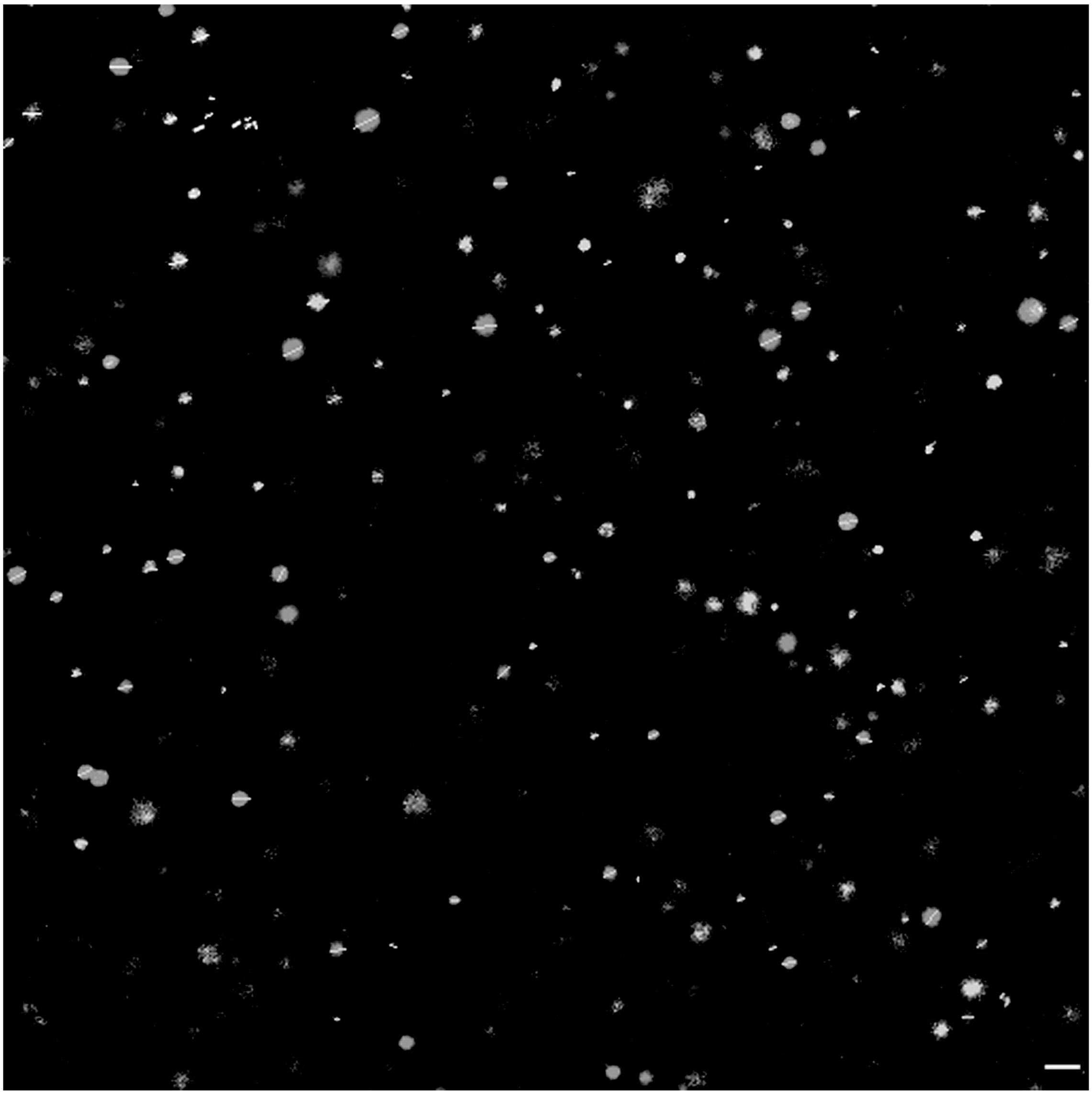

Protoplasts SYTO9-PI staining

- 100 protoplasts
- Mean: 2.2 µm
- SD 1.13 µm
- Median 1.9 µm

Supplementary Fig. 6. Protoplast formation and quantification. Scale bars correspond to 8 µm.

(B) Master images used to quantify protoplast diameter in *S. coelicolor* M145. Protoplasts were stained with PI and SYTO9. Protoplasts used for quantification are marked.

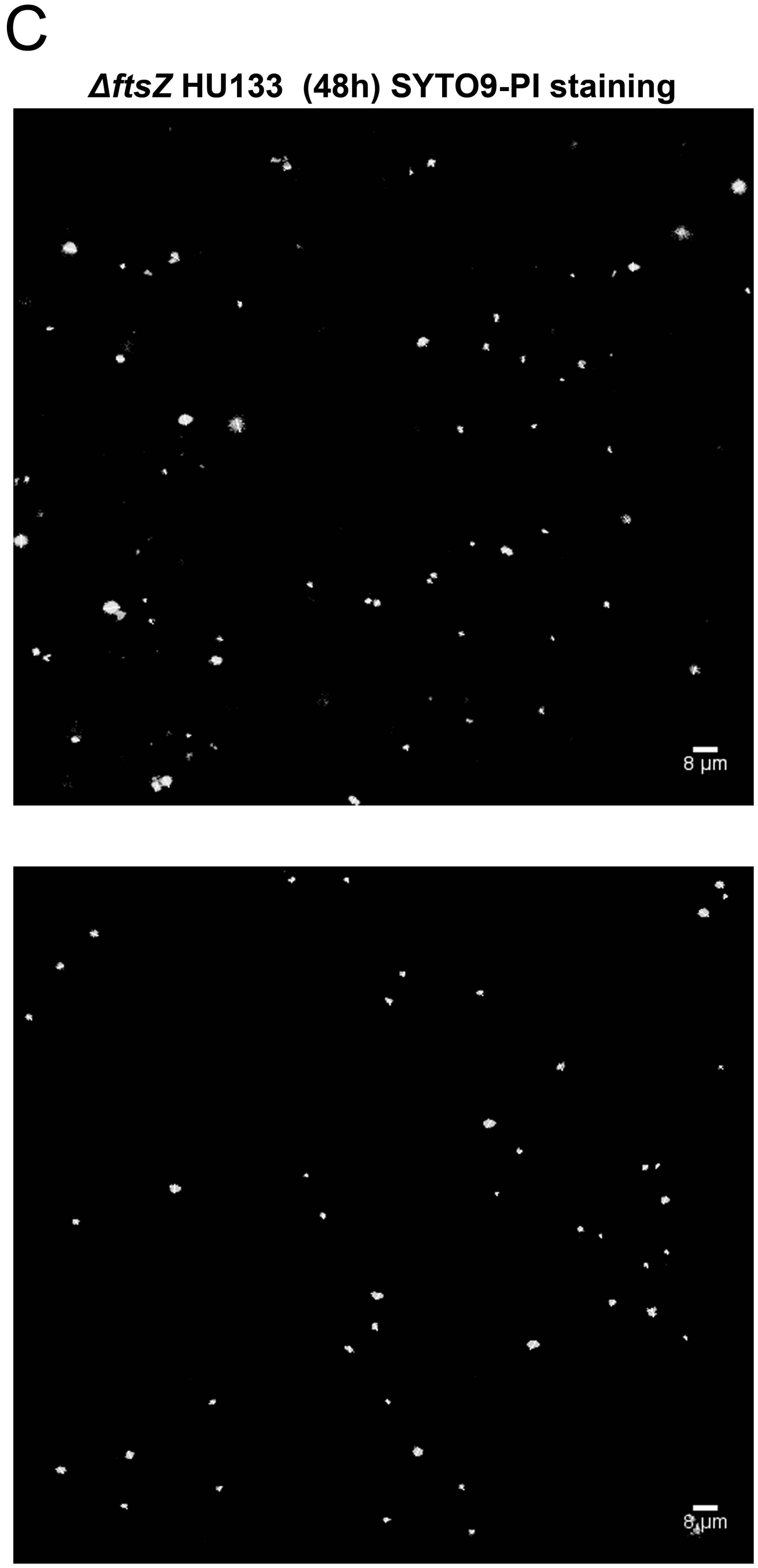

Protoplasts SYTO9-PI staining

- 100 protoplasts
- Mean: 1.98 µm
- SD 0.8 µm
- Median 1.81 µm

Supplementary Fig. 6. Protoplast formation and quantification. Scale bars correspond to 8 µm.

(C) Master images used to quantify protoplast diameter in *ΔftsZ* HU133. Protoplasts were stained with PI and SYTO9. Protoplasts used for quantification are marked.

Supplementary Table 1: TMT proteoimics.

(A) Peptides used for FtsZ protein abundance quantification. (B) FtsZ protein abundance. (C) FtsZ relative abundance (relative to 16-hours). R1-R4 Bilogical replicates 1-4.

(A) FtsZ peptides

FtsZ Peptides	TMT MI 16-hours				TMT MII 30-hours			TMT MII 65-hours		
	R1	R2	R3	R4	R1	R2	R3	R1	R2	R3
AAPQNYLAVIK	0.14	0.15	0.15	0.13	0.12	0.12	0.12	0.03	0.03	0.03
ANQAEDGIAELR	0.13	0.10	0.10	0.10	0.08	0.08	0.07	0.10	0.10	0.11
AVAAAEMAISSPLLEASIDGAR	0.11	0.10	0.11	0.11	0.10	0.09	0.09	0.10	0.09	0.09
DNVLGSSSAK	0.10	0.11	0.11	0.09	0.09	0.10	0.08	0.09	0.11	0.11
EEPEPAPVPEPVADLPVSPPPVPPSR	0.12	0.12	0.12	0.10	0.11	0.11	0.10	0.07	0.07	0.07
EEVDTLIVIPNDR	0.14	0.11	0.11	0.11	0.11	0.11	0.10	0.08	0.07	0.07
GADMVFVTAGEGGGTGTGGAPVVANIAR	0.11	0.12	0.11	0.10	0.12	0.11	0.12	0.08	0.07	0.06
GLGAGANPAVGR	0.11	0.11	0.11	0.10	0.10	0.10	0.09	0.08	0.09	0.09
RDNVLGSSSAK	0.10	0.12	0.10	0.10	0.10	0.10	0.09	0.09	0.10	0.10
SVMSEAGSALMGIGSAR	0.10	0.11	0.11	0.10	0.11	0.10	0.10	0.09	0.09	0.09
TYSDSAAEELDVPDFLK	0.14	0.14	0.13	0.12	0.12	0.12	0.12	0.04	0.05	0.04
VIGVGGGGVNAINR	0.13	0.12	0.12	0.11	0.11	0.10	0.10	0.07	0.06	0.07
VTVIAAGFDGGQPPSK	0.13	0.13	0.12	0.11	0.12	0.11	0.11	0.06	0.06	0.06

(B) FtsZ protein (53% sequence coverage)

Protein	TMT MI 16-hours				TMT MII 30-hours			TMT MII 65-hours			TMT abundance			q-values		
	R1	R2	R3	R4	R1	R2	R3	R1	R2	R3	MI 16h	MII 30	MII 65	MI16h vs MII65h	MI16h vs MII30h	MII30h vs MII65h
FtsZ	0.13	0.13	0.13	0.12	0.12	0.12	0.12	0.06	0.06	0.06	0.13	0.12	0.06	0.03	0.00	0.00

(C) FtsZ relative abundance (relative to 16-hours)

Sample	Time	TMT abundance			TMT ratios (relative to 16-hours)			FtsZ abundance	
		R1	R2	R3	R1	R2	R3	TMT average	TMT SD
MI 16-hours	16.00	0.13	0.13	0.13	1.00	1.00	1.00	1.00	0.00
MII 30-hours	30.00	0.12	0.12	0.12	0.90	0.90	0.90	0.90	0.00
MII 65-hours	65.00	0.06	0.06	0.06	0.49	0.44	0.43	0.45	0.03

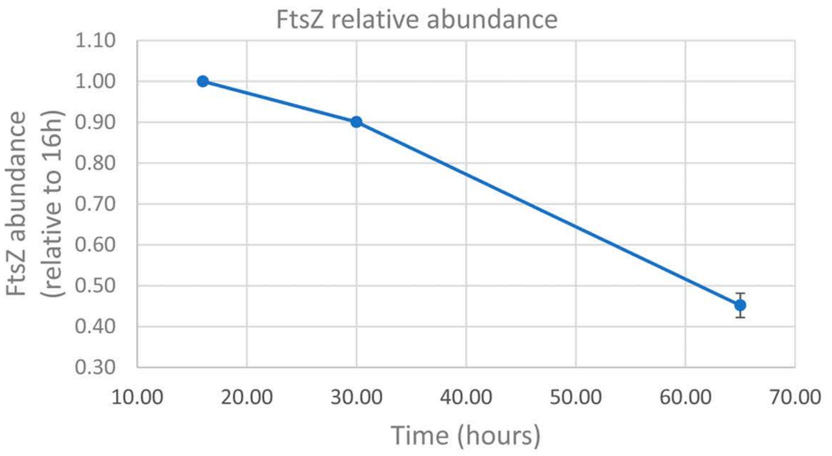

Supplement: Supplementary Information — Supplementary Figures 1-6, and Supplementary Table 1 [file ncomms12467-s1.pdf]
